# Supplementary material for: Rational highly dispersed ruthenium for reductive catalytic fractionation of lignocellulose
Source: Nat Commun. 2022 Aug 11;13:4716. doi: 10.1038/s41467-022-32451-5 (PMC9372153; doi:10.1038/s41467-022-32451-5)
Supplement: Supplementary file 1 — Supplementary Information [file 41467_2022_32451_MOESM1_ESM.pdf]

Supplementary Information for

Rational highly dispersed ruthenium for reductive catalytic fractionation of lignocellulose

Zhenzhen Liu[1], Helong Li[1], Xueying Gao[1], Xuan Guo[2], Shuizhong Wang[1]*, Yunming Fang[2]*, Guoyong Song[1]*

[1]Beijing Key Laboratory of Lignocellulosic Chemistry, Beijing Forestry University, Beijing, 100083, China

[2]College of Chemical Engineering, Beijing University of Chemical Technology, Beijing, 100029, China

*Email: S.W. szwang@bjfu.edu.cn, Y.F. fangym@mail.buct.edu.cn, G.S. songg@bjfu.edu.cn

Table of Contents

Supplementary Methods 2

Determination of Biomass Composition 11

Lignin-derived Products Analyses 12

Catalyst Recuperation and Carbohydrates Analyses 31

Reactivity of Lignin Model Compounds 36

Reported RCF Literatures 41

Standard Curves 44

Supplementary References 47

Supplementary Methods

Inductively Coupled Plasma–Atomic Emission Spectrometry (ICP–AES) was performed on IRIS Intrepid II XSP (Thermo Fisher) to determine the metal elemental of catalysts.

Brunauer–Emmett–Teller (BET) specific surface areas and pore size distribution were measured by N_2 adsorption-desorption isotherms at 77 K on a Quantachrome Autosorb-IQ system.

X-ray diffraction (XRD) patterns were characterized on Ultima IV using 3.0 kW with the Cu Kα radiation source.

Raman spectra were collected on a LabRAM ARAMIS Raman spectroscope equipped with a 532 nm laser source.

X-ray photoelectron spectroscopy (XPS) data were collected on Thermo Scientific K-Alpha electron spectrometer using an Al Kα radiation source.

High-angle annular dark-field scanning transmission electron microscopy (HAADF-STEM) images and corresponding elemental mapping were carried out on JEM-ARM300F with a spherical aberration corrector at 300 kV.

The X-ray absorption fine structure (XAFS) measurements of RuN/ZnO/C and RuN/ZnO/C-(R) were performed at Shanghai Synchrotron Radiation Facility (SSRF) and Beijing Synchrotron Radiation Facility (BSRF), respectively. The acquired EXAFS data were processed according to the standard procedures using the Athena and Artemis implemented in the IFEFFIT software packages. The fitting detail is described below according to reported literature[1]. The EXAFS spectra were obtained by subtracting the post-edge background from the overall absorption and then normalizing with respect to the edge-jump step. Subsequently, the $\chi(k)$ data of Ru K-edge were Fourier transformed to real (R) space using a Hanning windows (dk = 1.0 $Å^{-1}$) to separate the EXAFS contributions from different coordination shells. To obtain the quantitative structural parameters around central atoms, least-squares curve parameter fitting was performed

using the ARTEMIS module of IFEFFIT software packages[2]. The following EXAFS equation was used.

$$\chi(k) = \sum_j \frac{N_j S_0^2 F_j(k)}{k R_j^2} \exp[-2 k^2 \sigma_j^2] \exp[\frac{-2 R_j}{\lambda(k)}] \sin[2k R_j + \phi_j(k)] \qquad (1)$$

S_0^2 is the amplitude reduction factor, $F_j(k)$ is the effective curved-wave backscattering amplitude, N_j is the number of neighbors in the jth atomic shell, R_j is the distance between the X-ray absorbing central atom and the atoms in the jth atomic shell (backscatterer), λ is the mean free path in Å, $\phi_j(k)$ is the phase shift (including the phase shift for each shell and the total central atom phase shift), σ_j is the Debye-Waller parameter of the jth atomic shell (variation of distances around the average R_j). The functions $F_j(k)$, λ and $\phi_j(k)$ were calculated with the ab initio code FEFF8.2. The coordination numbers of model samples were fixed as the nominal values. The obtained S_0^2 was fixed in the subsequent fitting. While the internal atomic distances R, Debye Waller factor σ^2, and the edge-energy shift ΔE_0 were allowed to run freely.

Supplementary Table 1. ICP–AES analysis and N_2 adsorption-desorption for the RuN/ZnO/C catalyst.

Catalyst	ICP—AES analysis (wt%)		S_{BET} (m^2 g^{-1})	V_{pore} (cm^3 g^{-1})	D_{pore} (nm)
	Ru	Zn			
Fresh RuN/ZnO/C	0.12	6.2	688.6	0.5	3.9
Spent RuN/ZnO/C (2nd use)	0.12	4.9	231.3	0.3	3.9
Regenerated RuN/ZnO/C (3rd use)	0.11	4.5	564.8	0.5	3.9

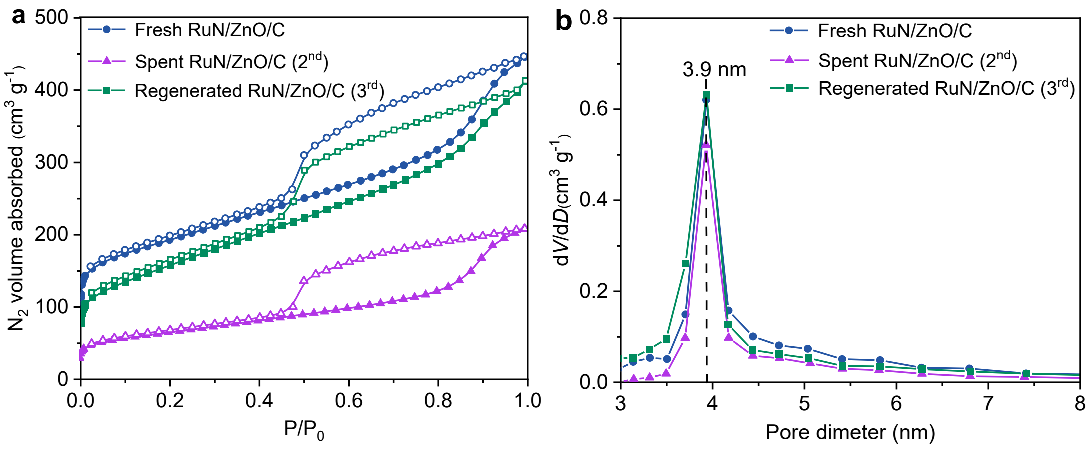

Supplementary Fig. 1 BET analyses of fresh, spent and regenerated RuN/ZnO/C catalysts. (**a**) N_2 adsorption-desorption isotherms, and (**b**) pore dimeter distribution plots for fresh, spent (2nd) and regenerated (3rd) RuN/ZnO/C catalysts.

Supplementary Note 1.

1) In the solution from fresh RuN/ZnO/C-catalyzed RCF of birch, Zn element was detected by ICP-AES analysis.

2) The spent RuN/ZnO/C (for 2nd use) was collected by filtration, and washing with MeOH before ICP-AES and N_2 adsorption-desorption analyses.

3) After 2nd use, the recovered catalyst was calcinated at 500 °C under N_2 flow for 2 h, thus giving regenerated RuN/ZnO/C (for 3rd use).

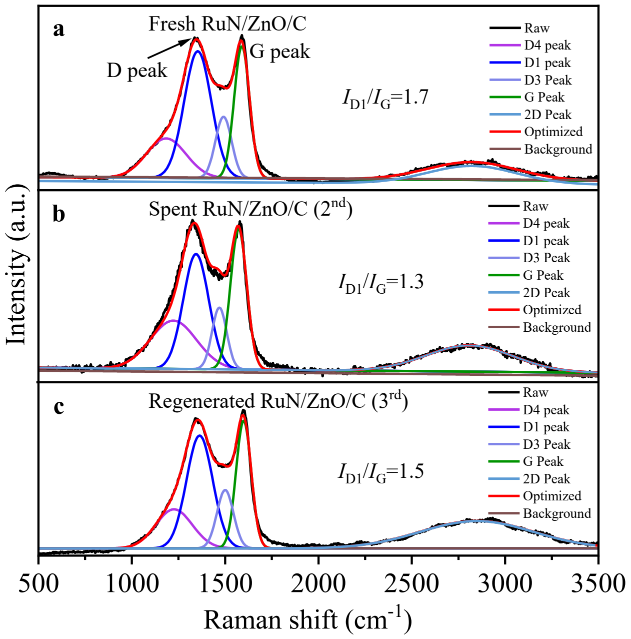

Supplementary Fig. 2 Raman spectroscopic studies. (**a**) fresh RuN/ZnO/C. (**b**) spent (2nd) RuN/ZnO/C. (**c**) regenerated (3rd) RuN/ZnO/C. a.u. refers arbitrary unit.

Supplementary Note 2. In Raman spectra of fresh RuN/ZnO/C, two characteristic Raman peaks around 1340 and 1593 cm^{-1} were associated with the vibrations of disordered sp^3 carbon (D peak) and hybridized graphitic sp^2 carbon (G peak), respectively. The D band was fitted into D1 (edge plane defects), D3 (amorphous carbon) and D4 (polyene-like architecture or introduction of ionic impurities) peaks. The D1/G intensity ratio (I_{D1}/I_G) was determined as 1.7, suggesting the presence of defects and disordered structures in the carbon support. The 2D peak of RuN/ZnO/C was observed at 2800 cm^{-1}, manifesting of graphene with few layers after the pyrolysis.

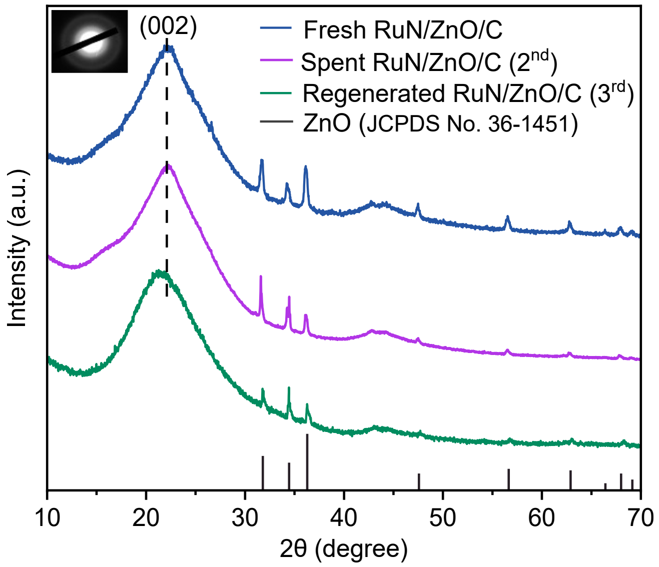

Supplementary Fig. 3 XRD patterns of fresh, spent (2nd) and regenerated (3rd) RuN/ZnO/C catalysts. The standard profile of ZnO (JCPDS No. 36-1451) is included in the bottom of figure. Inset: TEM image with SAED pattern of fresh RuN/ZnO/C catalyst. a.u. refers arbitrary unit.

Supplementary Note 3. The XRD pattern of fresh RuN/ZnO/C catalyst exhibited a broad diffraction peak centered at approximately 23°, corresponding to (002) plane of amorphous graphitic carbon. The selected area electron diffraction (SAED) pattern indicated that the fresh RuN/ZnO/C equipped with poor crystallinity.

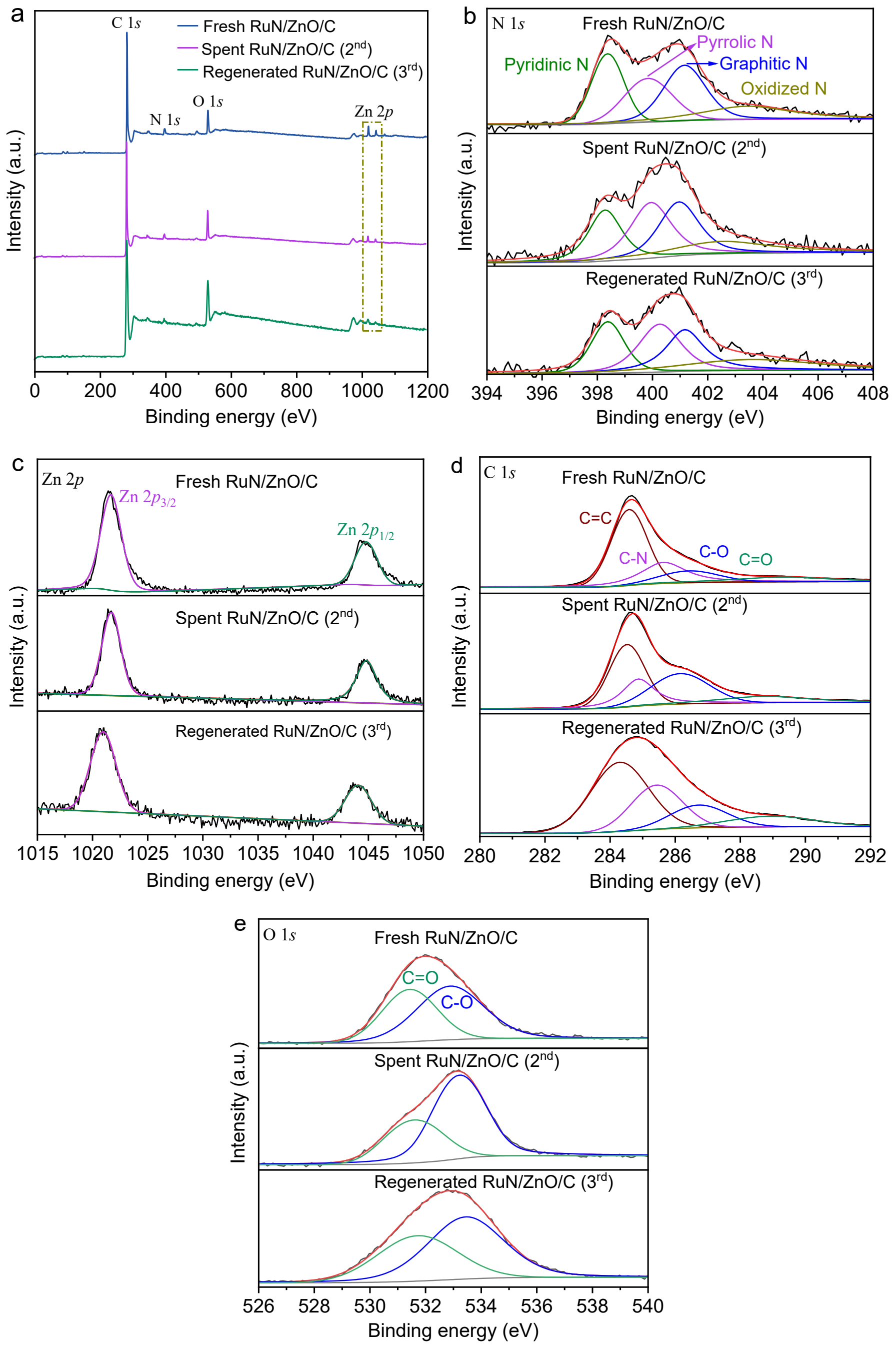

Supplementary Fig. 4 XPS analysis of fresh, spent and regenerated RuN/ZnO/C catalysts. (**a**) XPS spectra, high-resolution spectra of (**b**) N 1*s*, (**c**) Zn 2*p*, (**d**) C 1*s*, and (**e**) O1*s* for fresh, spent (2nd) and regenerated (3rd) RuN/ZnO/C catalysts. a.u. refers arbitrary unit.

Supplementary Note 4. In the C 1*s* high-resolution spectra (**d**), the peaks at 284.7 eV, 285.6 eV, 286.4 eV and 289.3 eV were assigned to C=C, C-N, C-O and C=O, respectively. There were two bonds corresponding to C=O and C-O groups in the O 1*s* high-resolution spectra (**e**).

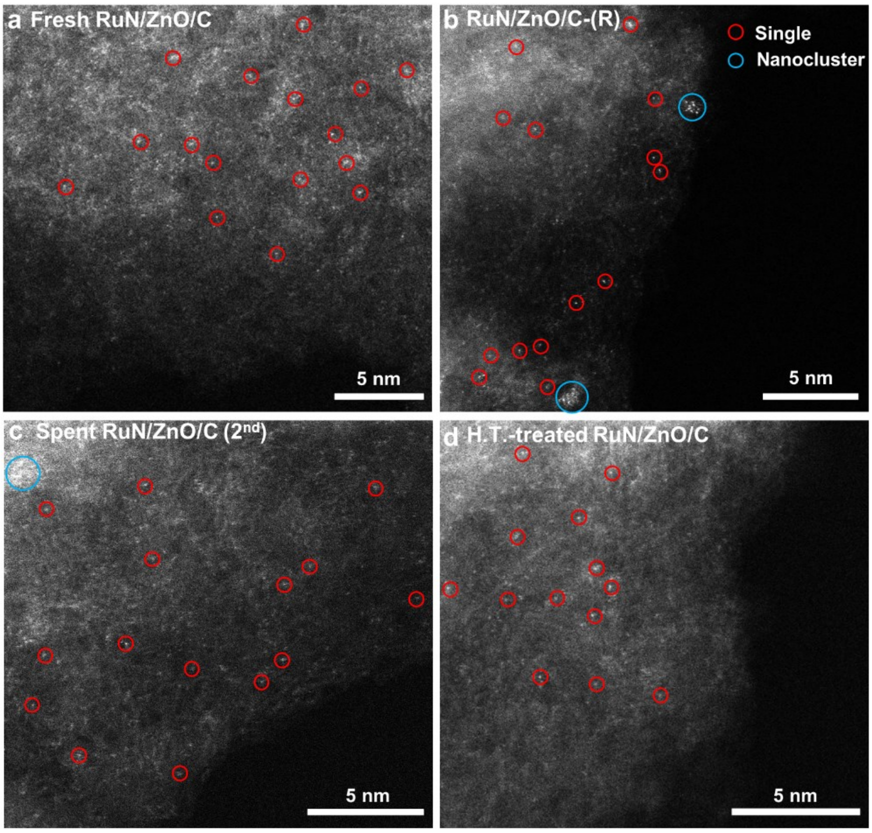

Supplementary Fig. 5 HAADF-STEM images of different catalysts. (**a**) fresh RuN/ZnO/C, (**b**) RuN/ZnO/C-(R), (**c**) spent RuN/ZnO/C (2nd) catalyst, and (**d**) H.T.-treated RuN/ZnO/C.

Supplementary Note 5. In HAADF-STEM images, the RuN/ZnO/C-(R) and spent RuN/ZnO/C catalysts exhibited both atomically dispersed Ru single atoms and Ru nanoclusters.

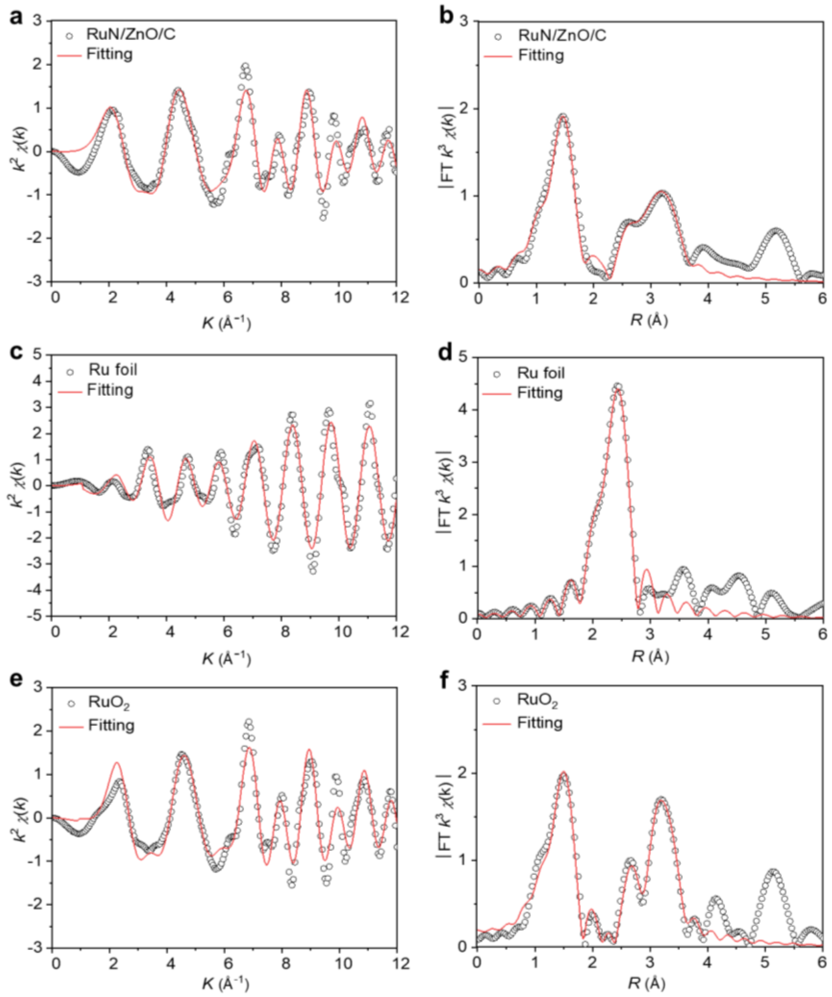

Supplementary Fig. 6 XAFS fitting curves. (**a**) k space EXAFS of RuN/ZnO/C, (**b**) FT-EXAFS of RuN/ZnO/C, (**c**) k space EXAFS of Ru foil, (**d**) FT-EXAFS of Ru foil, (**e**) k space EXAFS of RuO_2, and (**f**) FT-EXAFS of RuO_2.

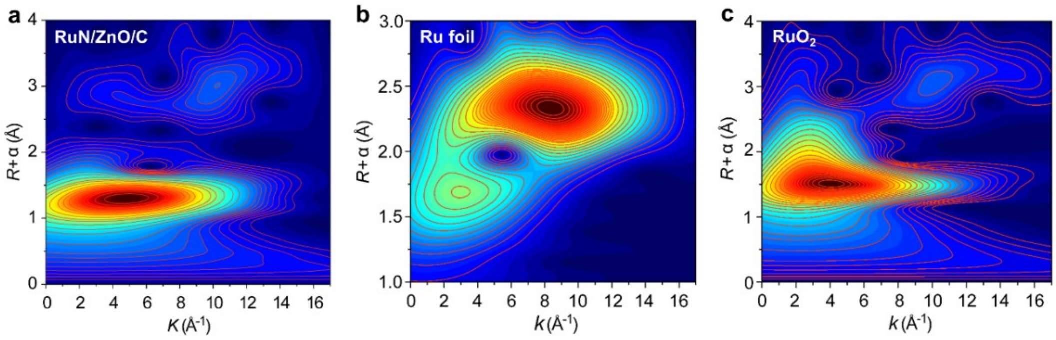

Supplementary Fig. 7 Wavelet transformed (WT) k^2-weighted $\chi(k)$-function of the Ru-K edge EXAFS spectra. (**a**) RuN/ZnO/C, (**b**) Ru foil, and (**c**) RuO_2.

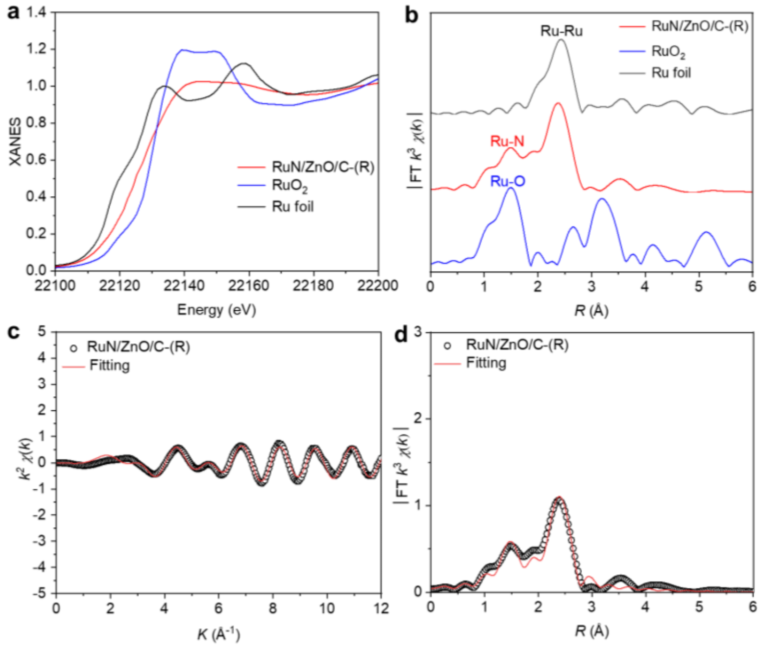

Supplementary Fig. 8 XAFS data of RuN/ZnO/C-(R). (**a**) Ru K-edge XANES and (**b**) FT-EXAFS of RuN/ZnO/C-(R), (**c**) k space EXAFS and (**d**) FT-EXAFS fitting curves of RuN/ZnO/C-(R).

Supplementary Table 2. Structural parameters extracted from the Ru K-edge EXAFS fitting. (S_0^2=0.679)

Samples	Scattering pair	*CN*	*R* (Å)	σ^2 (10^{-3}Å^2)	ΔE_0 (eV)	*R* factor
Ru foil	Ru-Ru	12*	2.67±0.01	1.75±0.5	0.68±0.66	0.012
RuO_2	Ru-O	3.6±0.3	1.94±0.01	1.5±0.8	-2.86±1.44	0.014
RuN/ZnO/C	Ru-N	4.2±0.7	1.95±0.01	3.03±1.8	-6.34±2.7	0.02
RuN/ZnO/C-(R)	Ru-N	1.7±0.4	1.95±0.01	4±1	-3.84±2.17	0.02
	Ru-Ru	3.0±0.8	2.67±0.01	4±1	-3.84±2.17	

Supplementary Note 6. S_0^2 is the amplitude reduction factor; *CN* is the coordination number; *R* is interatomic distance (the bond length between central atoms and surrounding coordination atoms); σ^2 is Debye-Waller factor (a measure of thermal and static disorder in absorber-scatter distances); ΔE_0 is edge-energy shift (the difference between the zero kinetic energy value of the sample and that of the theoretical model). *R* factor is used to value the goodness of the fitting.

Determination of Biomass Composition

Biomass compositions were determined according to standard analytical procedure (NREL/TP-510-42618)[3]. The biomass chips (60-mesh) were extracted with toluene/ethanol (2:1, v/v) in a Soxhlet instrument for 12 h, and then dried at 80 °C for 5 h. The mixture containing 300 mg of dried biomass and 3 mL of 72 wt% H_2SO_4 solution were stirred at 30 °C for 2 h, which was then diluted by adding 84 mL deionized water, and heated at 120 °C for 1 h in the high-pressure sterilizer. After the reactor was cooled to room temperature, the solid residue and solution were separated through filtration. The solid residue was dried at 110 °C to gain Klason lignin (AIL). The acid soluble lignin (ASL) in filtrate was analyzed using UV spectra by recording the absorbance of the soluble fraction at 240 nm. Finally, the determination of carbohydrates in the aqueous soluble fraction was performed on HPLC system.

Supplementary Table 3. Compositional analysis of various lignocellulosic biomass.

Samples	AIL (wt%)	ASL (wt%)	Cellulose (wt%)	Hemicellulose (wt%)	Extraction (wt%)	Total (wt%)
Birch	22.7	2.6	42.3	19.6	3.5	90.7
Beech	24.6	1.7	40.1	18.2	5.6	90.2
Eucalyptus	23.8	3.2	41.6	16.9	3.2	88.7
Poplar	20.0	1.5	50.4	18.6	3.2	93.7
Pine	31.1	0.9	47.1	9.3	4.3	92.7
Spruce	34.9	1.2	45.3	8.6	4.5	94.5
Miscanthus	14.6	2.6	48.7	21.6	2.2	89.7

Lignin-derived Products Analyses

After RCF reaction, the soluble fraction containing lignin-derived products and insoluble fraction containing carbohydrate and catalyst were separated though filtration with a 0.22 μm filter. The soluble faction was evaporated and extracted using dichloromethane/water, thereby giving orange-brown lignin oils after the removal of organic solvents. The qualitative and quantitative analyses of lignin oils were performed on GC-MS (Shimadzu GC 2010 series equipped with a HP-5 MS column and a mass spectroscopy detector) and GC (Shimadzu GC 2010 series equipped with a HP-5 column and a flame ionization detector (FID)). The following GC or GC-MS procedure was used: 1 μL aliquot with a split ratio of 20:1, injection temperature of 250 °C, column temperature program: 50 °C (hold time 3 min), 8 °C min^{-1} to 280 °C (hold time 5 min), detection temperature of 290 °C (for FID) or 280 °C (for MS). The quantification of monomers in the lignin oily samples were evaluated with authentic samples by using commercially available standards or independently synthesized standards. The as-obtained lignin oils were diluted to 10 mg/mL in ethyl acetate containing internal standard (tetradecane) before GC and GC-MS systems. Linear calibration curves were produced from 0.14 mg/mL to 3.9 mg/mL for authentic samples.

The lignin oil yields were calculated based on the Klason lignin weight, calculating formula as shown below:

$$\text{Phenolic monomers (wt\%)}=\frac{\text{Mass (total monomers)}}{\text{Mass (Klason lignin)}}\times 100\% \quad (2)$$

The degrees of delignification, and the retentions of cellulose and hemicellulose in pulps were calculated based on the biomass compositional analyses of solid pulps (the catalyst has been removed) [3]. Calculating formula as shown below:

$$\text{Delignification (wt\%)}=\frac{\text{Mass (Klason lignin)-Mass (lignin in pulp)}}{\text{Mass (Klason lignin)}}\times 100\% \quad (3)$$

$$\text{Sugar retention (wt\%)}=\frac{\text{Mass (Sugar in pulp)}}{\text{Mass (Sugar in sawdust)}}\times 100\% \quad (4)$$

To identify the probable dimers, the lignin oils (*ca*. 50 mg) were dissolved in anhydrous tetrahydrofuran (5 mL), to which *N,O*-bis(trimethylsilyl)trifluoroacetamide (BSTFA) (250 μL) was added. The mixture was stirred at 65 °C for 1 h under N_2, which was then analyzed on GC-MS. The following GC-MS procedure was used: 1 μL aliquot with a split ratio of 50:1, injection temperature of 250 °C, column temperature program: 50 °C (hold time 3 min), 5 °C min^{-1} to 280 °C (hold time 5 min), detection temperature of 280 °C. The probable structures of dimers were identified based on mass spectra analyses and reported literatures[4-6].

To obtain the 2D HSQC NMR spectra, the lignin oil (100 mg) was dissolved in 0.7 mL of DMSO-d_6, which was then recorded on a Bruker Avance 400 MHz spectrometers. The solvent peak (δ_C = 39.5, δ_H = 2.49 ppm) was used as an internal reference. HSQC experiments had the following parameters: acquiring from 15 to 0 ppm in F2 (^{1}H) by using 2048 data points for an acquisition time (AQ) of 128 ms, 210-0 ppm in F1 (^{13}C) by using 512 increments (F1 acquisition time 11.6 ms) of 48 scans with a 1.5 s interscan delay (D1). HSQC cross-peaks were assigned by comparing the spectra with the authentic monomeric and dimeric samples.

The average molecular weight of the lignin-derived products was investigated using gel permeation chromatography (GPC). The as-obtained lignin oils were dissolved in THF to form solutions (2 mg mL^{-1}), which were then filtered with 0.22 μm filter to remove any particle matters. GPC analyses were performed on Shimadzu LC-20AD equipped with a PLgel 3 μm Mixed-E column and UV detection at 254 nm, using THF as the mobile phase (1 mL/min). The system was calibrated using Polystyrene standards with different molecular weights (162 g mol^{-1}, 370 g mol^{-1}, 580 g mol^{-1}, 860 g mol^{-1}, and 1320 g mol^{-1}, Polystyrene Calibration kit S-L2-10, Part Number PL 2010-0105, Agilent Technologies).

Supplementary Table 4. Products distribution from RCF reaction of birch chips with different catalyst.[a]

Catalyst	Phenolic monomers yield (wt%)							Selectivity (**Pr–G**, **Pr–S**) (mol%)	TON[b]	S/G	M_w (g/mol)	Delignification (wt%)	Sugar retention (wt%)	
	**Pr-G**	**Pe-G**	**Pr–S**	**Pe-S**	**POH–S**	Others	Total						C5	C6
None	0.6	1.0	0.4	2.3	N.D.[c]	2.2	6.5	17.2	-	1.1	921	51	70	95
RuN/C	6.0	0.9	11.4	3.4	0.7	2.5	24.9	69.9	240	1.8	505	83	75	93
RuN/C + $Zn(OAc)_2$[d]	3.2	1.0	3.8	5.2	0.6	7.6	21.4	33.1	209	1.9	510	80	60	88
RuN/ZnO/C	11.1	N.D.	27.9	N.D.	3.5	3.9	46.4	84.7	431	2.4	325	95	72	96
RuN/ZnO/C-(R)[e]	10.7	N.D.	27.4	N.D.	3.2	4.8	46.1	83.0	429	2.5	314	95	73	97
ZnO/C	1.1	0.9	0.6	2.5	N.D.	3.0	8.1	20.4	N.D.	2.1	919	73	49	76
Ru/ZnO/C-(MOF)	1.6	5.6	2.4	17.2	3.0	5.2	35.0	11.8	204	2.4	-	96	73	92
H.T.-treated Ru/ZnO/C-(MOF)	0.9	4.7	1.1	15.1	2.8	5.4	30.0	6.7	174	2.3	-	95	72	90
Ru/C[f]	8.6	N.D.	20.2	N.D.	13.6	4.3	46.7	63.8	22	2.5	404	95	59	93
Ru/C[g]	5.9	N.D.	12.2	N.D.	13.3	5.7	37.1	53.3	45	2.3	512	-	-	-

[a] Reaction conditions: birch wood (250 mg), catalyst (25 mg, 10 wt%) or no catalyst, MeOH (15 mL), 240 °C, H_2 (3 MPa at 25 °C), and 4 h. [b] TON denotes turnover numbers, calculated based on the total number of moles of Ru in the catalyst ($mol_{phenols}\ mol_{Ru}^{-1}$). [c] N.D. refers to not detected. [d] Reaction conditions: birch wood (250 mg), RuN/C (25 mg, 10 wt%), $Zn(OAc)_2$ (5 mg), MeOH (15 mL), 240 °C, H_2 (3 MPa at 25 °C), and 4 h. [e] RuN/ZnO/C-(R) was obtained from the pre-reduction of RuN/ZnO/C by H_2 (3 MPa at 25 °C) in MeOH at 240 °C for 4 h. [f, g] 12.5 mg (5 wt%) and 5 mg (2 wt%) of commercial Ru/C were used, respectively.

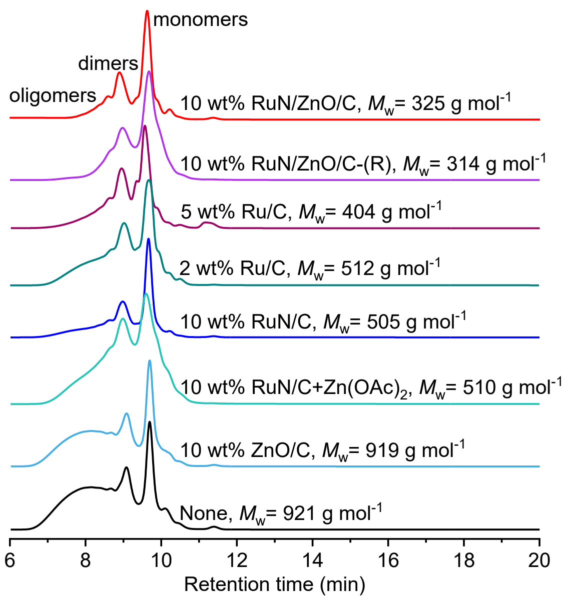

Supplementary Fig. 9 GPC spectra of lignin-derived products with different catalysts. Reaction conditions: birch wood (250 mg), catalyst or no catalyst, MeOH (15 mL), 240 °C, H_2 (3 MPa at 25 °C), and 4 h.

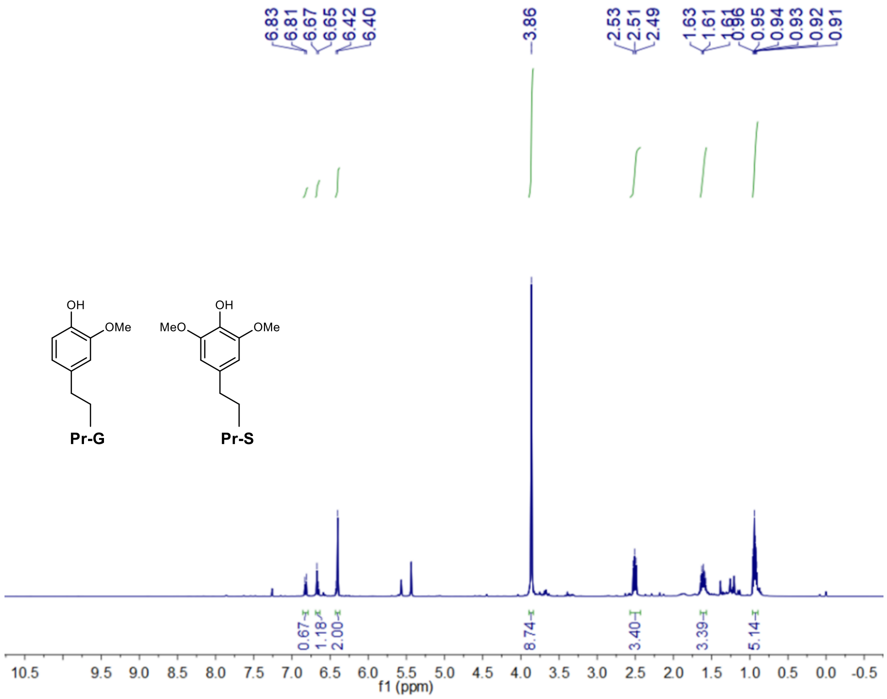

Supplementary Fig. 10 ^{1}H NMR spectrum of mixture of Pr-G and Pr-S after short column chromatography. The as-obtained oily product was purified through chromatographic column (PE/EA=4/1) to produce mixture of Pr-S and Pr-G (89 mg per gram of birch biomass).

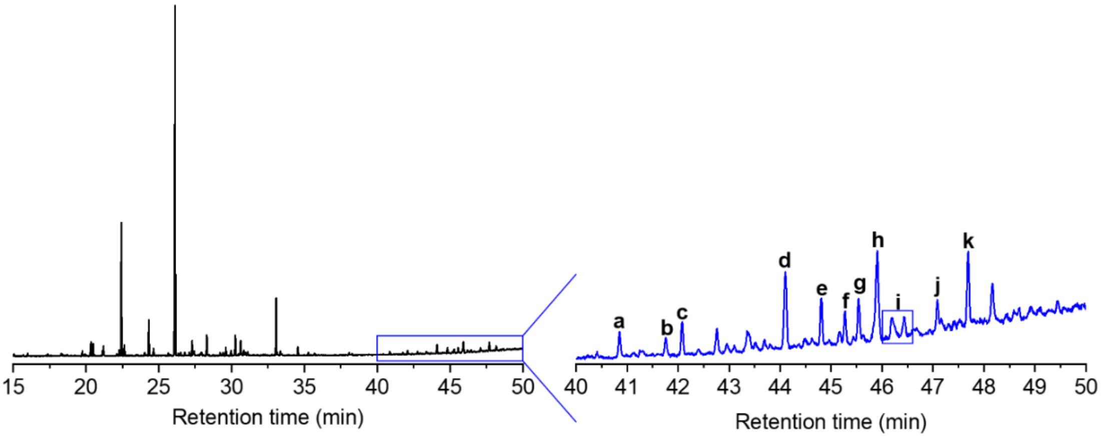

Dimer **a**. Retention time = 40.854 min

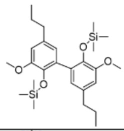

Molecular weight: 474.79

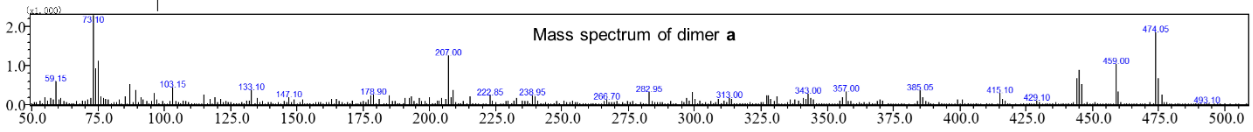

Dimer **b**. Retention time = 41.755 min

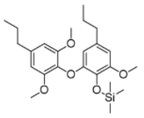

Molecular weight: 432.63

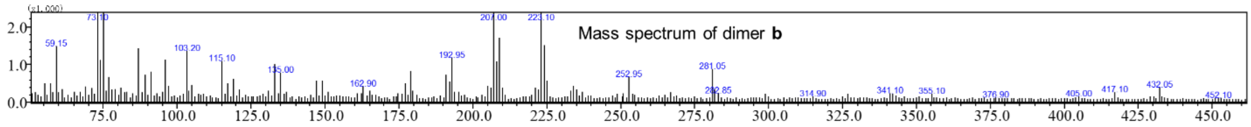

Dimer **c**. Retention time = 42.074 min

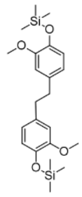

Molecular weight: 418.68

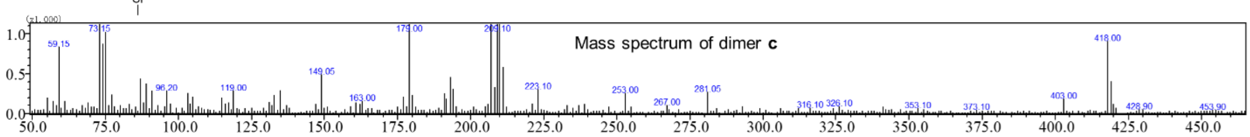

Dimer **d**. Retention time = 44.109 min

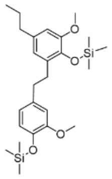

Molecular weight: 460.76

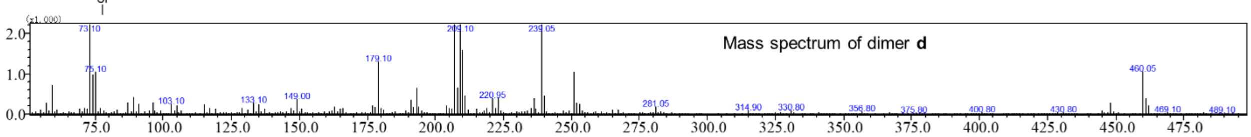

Dimer **e**. Retention time = 44.817 min

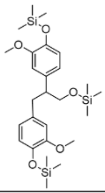

Molecular weight: 520.89

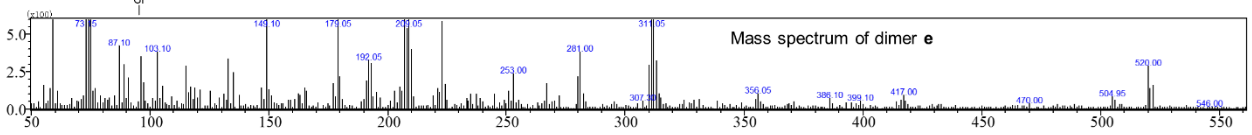

Dimer **f**. Retention time = 45.276 min

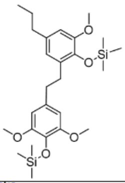

Molecular weight: 490.79

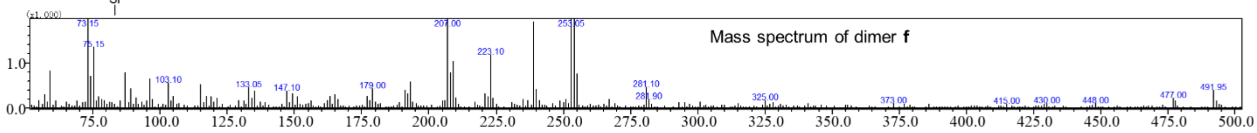

Dimer **g**. Retention time = 45.551 min

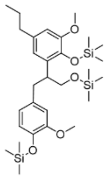

Molecular weight: 562.97

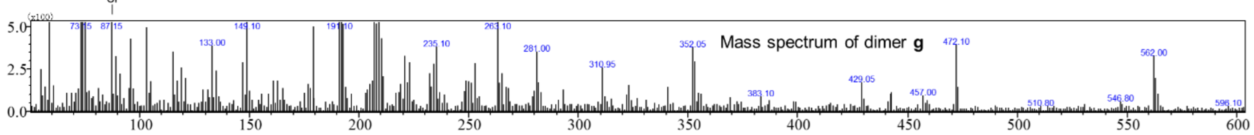

Dimer **h**. Retention time = 45.907 min

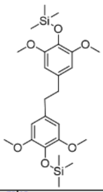

Molecular weight: 478.73

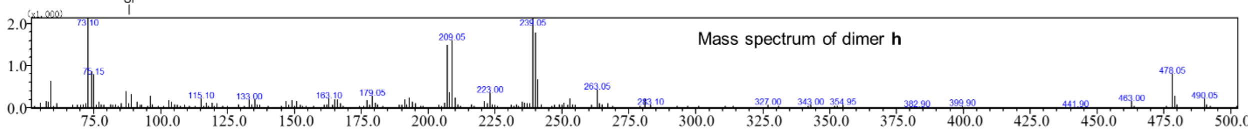

Dimer **i**. Retention time = 46.192 min, 46.437 min

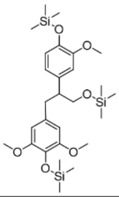

Molecular weight: 550.91

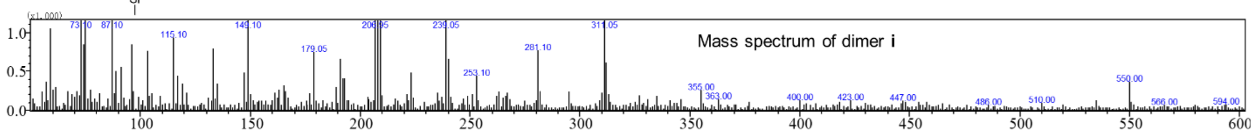

Dimer **j**. Retention time = 47.088 min

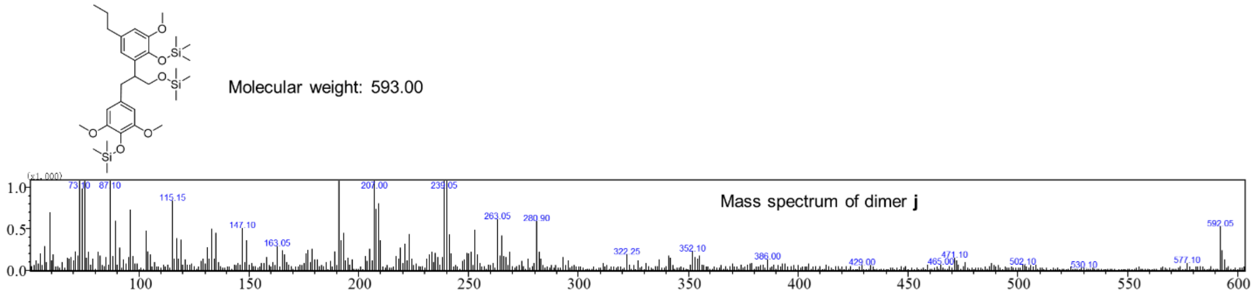

Dimer **k**. Retention time = 47.699 min

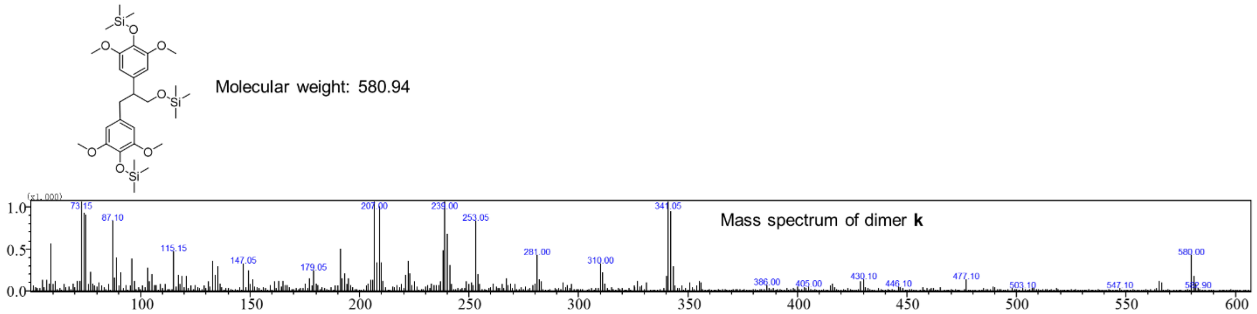

Supplementary Fig. 11 The information on the possible dimers of the lignin oils.

GC-MS spectra of silylated lignin oils and structure analyses for possible dimers.

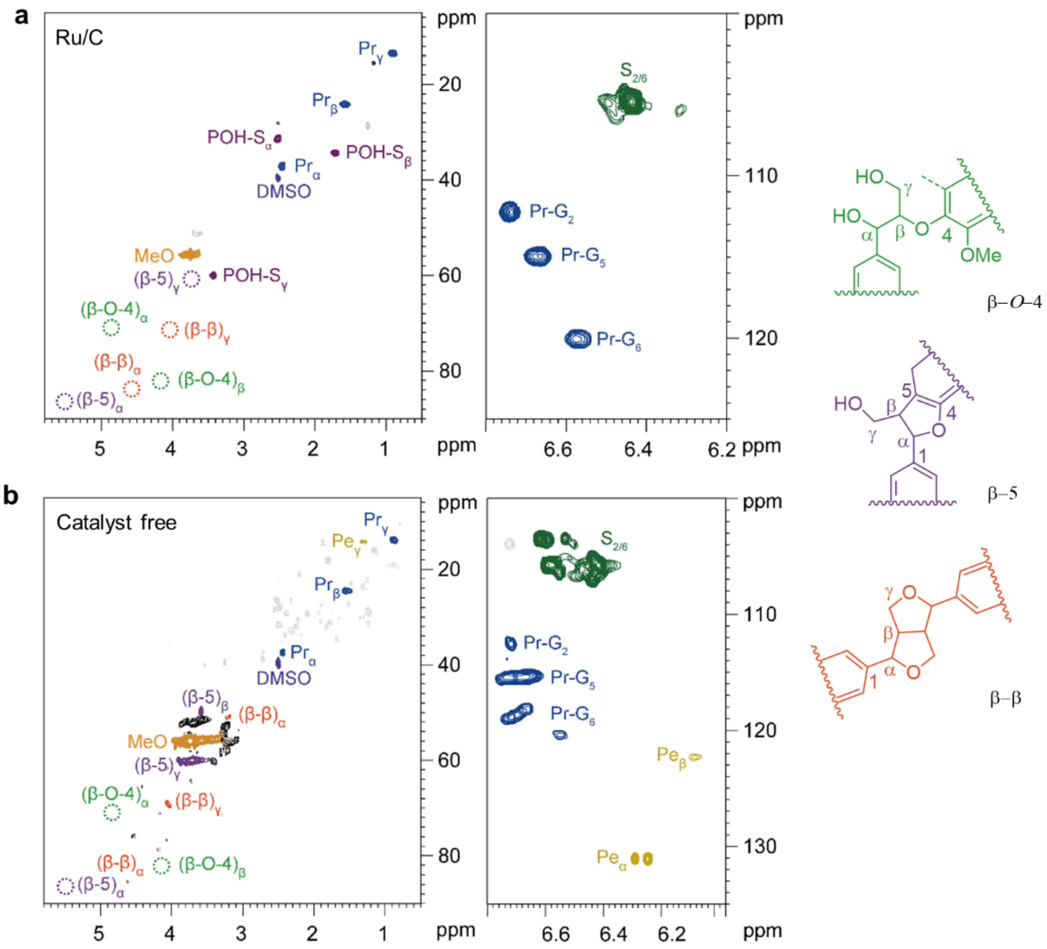

Supplementary Fig. 12 2D HSQC NMR spectra of lignin oily products (DMSO-*d*₆) from RCF of birch. (**a**) 5 wt% of Ru/C catalyst, (**b**) catalyst free conditions.

Supplementary Table 5. Products distribution of RuN/ZnO/C-catalyzed RCF reaction of birch chips with different solvents.[a]

Solvent	Phenolic monomers yield (wt%)							Selectivity (**Pr–G, Pr–S**) (mol%)	Selectivity (**Pe–G, Pe–S**) (mol%)	TON[b]	S/G	M_w (g/mol)	Delignification (wt%)
	**Pr-G**	**Pe-G**	**Pr–S**	**Pe-S**	**POH–S**	Others	Total						
MeOH	8.5	1.3	16.2	8.4	2.4	1.3	38.1	66.2	25.3	359	2.4	341	89
EtOH	6.7	1.5	10.2	8.7	2.3	2.4	31.8	54.2	33.0	303	2.4	327	72
^{i}PrOH	4.8	1.7	8.4	7.1	1.1	1.5	24.6	54.1	35.9	235	2.0	320	61
THF	2.5	2.1	4.5	5.5	0.7	1.2	16.5	42.7	46.1	159	1.7	343	55
Dioxane	1.7	2.6	2.4	6.1	0.6	1.1	14.5	28.2	59.8	140	1.7	434	43

[a] Reaction conditions: birch wood (250 mg), RuN/ZnO/C (25 mg, 10 wt%), solvent (15 mL), 220 °C, H_2 (3 MPa at 25 °C), and 4 h. [b] TON denotes turnover numbers, calculated based on the total number of moles of Ru in the catalyst ($mol_{phenols}\ mol_{Ru}^{-1}$).

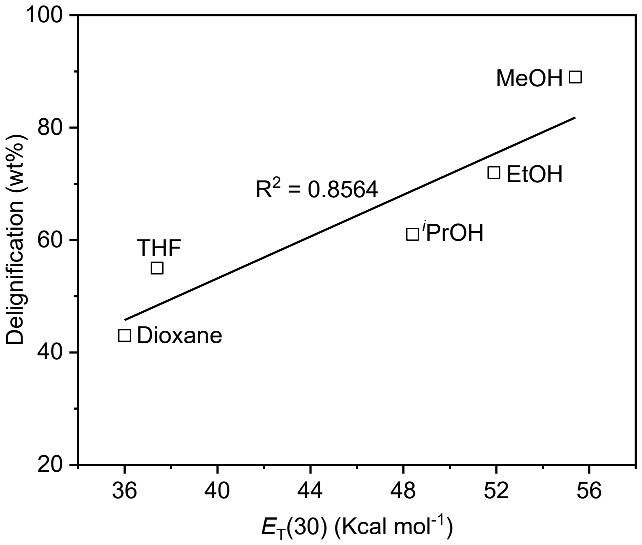

Supplementary Fig. 13 The relationship between the degrees of delignification and solvent polarity (E_T(30)). The degrees of delignification approximately follow linear relationships with the solvent polarity.

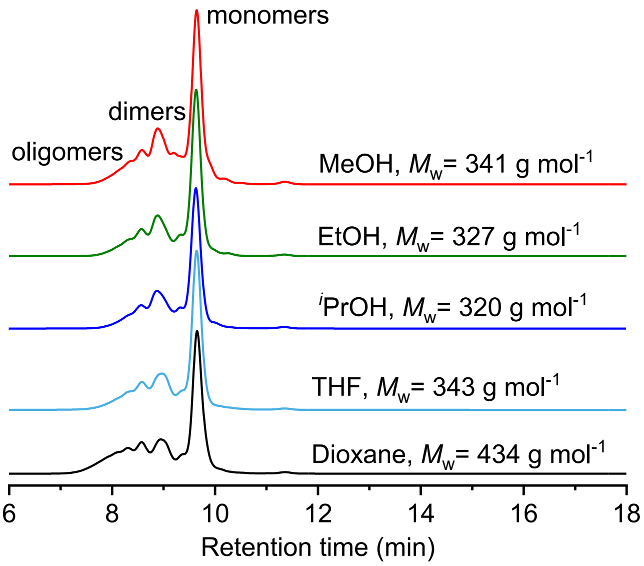

Supplementary Fig. 14 GPC spectra of lignin-derived products in different solvents. Reaction conditions: birch wood (250 mg), RuN/ZnO/C (25 mg, 10 wt%), solvent (15 mL), 220 °C, H_2 (3 MPa at 25 °C), and 4 h.

Supplementary Table 6. Products distribution of RuN/ZnO/C-catalyzed RCF reaction of birch at different reaction temperatures.[a]

Temp. (°C)	Phenolic monomers yield (wt%)[b]							Selectivity (Pr–G, Pr–S) (mol%)	Selectivity (Pe–G, Pe–S) (mol%)	TON[b]	S/G	M_w (g/mol)
	**Pr-G**	**Pe-G**	**Pr–S**	**Pe-S**	**POH–S**	Others	Total					
160	0.7	1.5	0.5	2.5	0.2	2.0	7.4	15.8	69.5	72	1.1	396
180	2.7	3.1	2.7	7.9	1.4	4.1	21.9	25.5	59.9	212	1.7	375
200	4.6	3.4	5.8	9.3	1.5	3.5	28.1	37.8	52.2	270	1.8	369
220	8.5	1.3	16.2	8.4	2.4	1.3	38.1	66.2	25.4	359	2.4	341
240	11.1	N.D.[c]	27.9	N.D.	3.5	3.9	46.4	84.7	0	431	2.4	325
260	10.3	N.D.	24.6	N.D.	2.2	6.1	43.2	80.1	0	416	2.2	394

[a] Reaction conditions: birch wood (250 mg), RuN/ZnO/C (25 mg, 10 wt%), MeOH (15 mL), H_2 (3 MPa at 25 °C), and 4 h. [b] TON denotes turnover numbers, calculated based on the total number of moles of Ru in the catalyst ($mol_{phenols}\ mol_{Ru}^{-1}$). [c] N.D. refers to not detected.

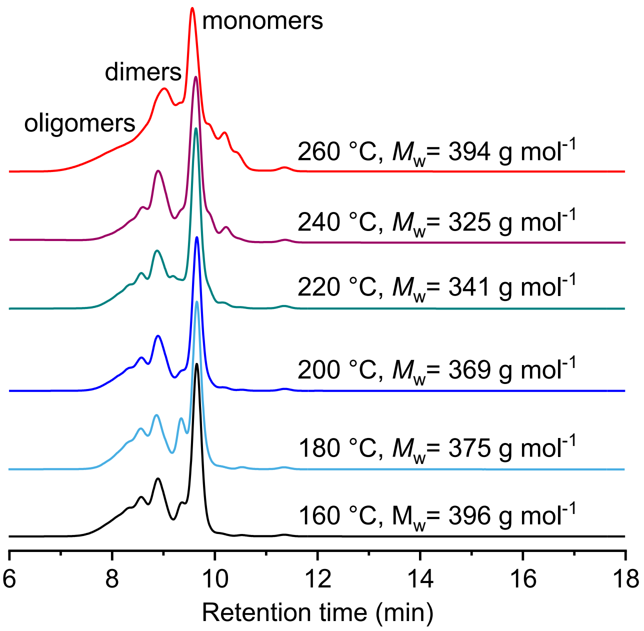

Supplementary Fig. 15 GPC spectra of lignin-derived products at different reaction temperatures. Reaction conditions: birch wood (250 mg), RuN/ZnO/C (25 mg, 10 wt%), MeOH (15 mL), H_2 (3 MPa at 25 °C), and 4 h.

Supplementary Table 7. Products distribution of RuN/ZnO/C-catalyzed RCF reaction of birch chips under different H_2 pressure.[a]

Pressure (R.T.) (MPa)	Pressure (220 °C) (MPa)	Phenolic monomers yield (wt%)							Selectivity (Pr–G, Pr–S) (mol%)	Selectivity (Pe–G, Pe–S) (mol%)	TON[b]	S/G	M_w (g/mol)
		**Pr-G**	**Pe-G**	**Pr–S**	**Pe-S**	**POH–S**	Others	Total					
0.1 N_2	4.6	0.1	3.6	N.D.[c]	4.8	0.2	1.6	10.3	1.4	96.9	103	0.9	636
0.1 H_2	5.0	2.2	4.6	2.6	10.1	0.5	3.1	23.1	20.9	74.7	223	1.6	442
1 H_2	6.6	3.6	4.6	4.5	13.1	0.6	4.2	30.6	26.9	65.7	295	1.8	383
3 H_2	8.6	8.5	1.3	16.2	8.4	2.4	1.3	38.1	66.2	25.4	359	2.4	341
4 H_2	10.0	9.5	0.7	22.4	4.0	3.1	2.3	42.0	76.9	13.9	396	2.3	319

[a] Reaction conditions: birch wood (250 mg), RuN/ZnO/C (25 mg, 10 wt%), MeOH (15 mL), 220 °C, and 4 h. [b] TON denotes turnover numbers, calculated based on the total number of moles of Ru in the catalyst ($mol_{phenols}\ mol_{Ru}^{-1}$). [c] N.D. refers to not detected

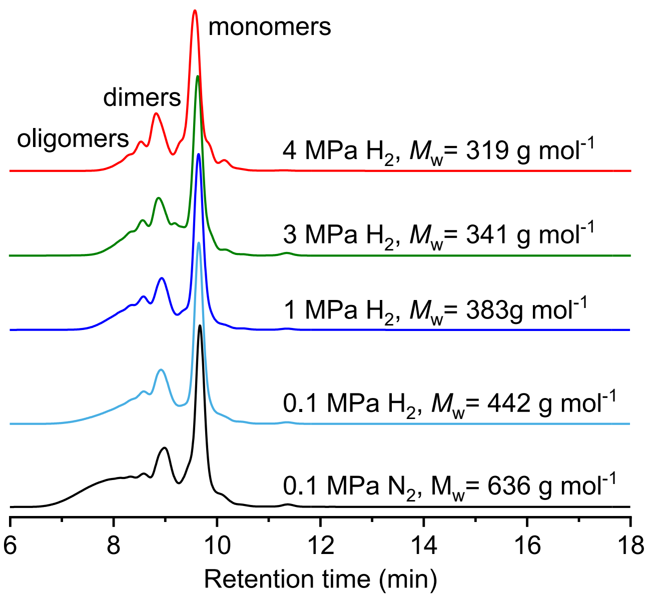

Supplementary Fig. 16 GPC spectra of lignin-derived products under different H_2 pressure. Reaction conditions: birch wood (250 mg), RuN/ZnO/C (25 mg, 10 wt%), MeOH (15 mL), 220 °C, and 4 h.

Supplementary Table 8. Thermodynamic properties of different H_2 pressure and temperature in MeOH solvent.

	Temp. (K)	P (H_2 MPa) (R.T.)	P (H_2 MPa) (Final)	State of MeOH	H_2 solubility (mole fraction)
MeOH, 15 mL In a 50 mL reactor	493.15	0.1	5.0	gas	0.0422
	493.15	1	6.6	liquid	0.0455
	493.15	3	8.6	liquid	0.0489
	493.15	4	10.0	liquid	0.0510
	433.15	3	4.4	liquid	0.0282
	453.15	3	6.2	liquid	0.0352
	473.15	3	7.3	liquid	0.0416
	513.15	3	12.4	liquid	0.0606
	533.15	3	13.8	supercritical phase	0.0694
MeOH, 150 mL In a 300 mL reactor	473.15	3	7.2	liquid	0.0415

Supplementary Note 7. Mole fraction solubility of H_2 in MeOH was calculated by the reported literature[7]. The equation of H_2 solubility was expressed as x = $0.053643 \cdot N^{0.427151} \cdot T_r^{2.827265} \cdot P_r^{0.274070}$, where, N is the number of carbon atoms of MeOH ($N = 1$), T_r and P_r are the reduced temperature ($T_r = T/T_c$, $T_c = 512.5$ K) and pressure ($P_r = P/P_c$, $P_c = 8.084$ MPa).

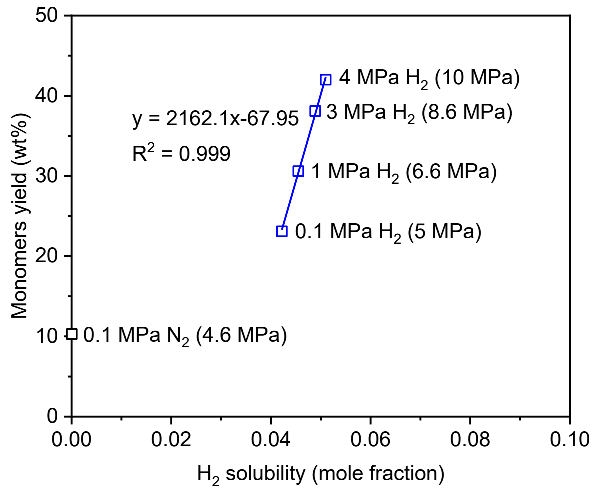

Supplementary Fig. 17 The relationship of mole fraction H_2 solubility and monomers yield. The values of pressure in parentheses refer the final pressure at 220 °C.

Supplementary Table 9. Products distribution of RuN/ZnO/C-catalyzed RCF reaction of birch chips with different catalyst dosage.[a]

Catalyst Dosage (wt%)	Phenolic monomers yield (wt%)							Selectivity (Pr-G, Pr-S) (mol%)	Selectivity (Pe–G, Pe–S) (mol%)	TON[b]	S/G	M_w (g/mol)
	**Pr-G**	**Pe-G**	**Pr–S**	**Pe-S**	**POH–S**	Others	Total					
None	0.4	0.8	N.D.[c]	1.3	N.D.	3.8	6.3	-	-	-	1.2	921
5	3.2	4.8	3.3	11.6	1.1	3.3	27.3	24.3	69.4	524	1.7	427
10	8.5	1.3	16.2	8.4	2.4	1.3	38.1	66.2	25.4	359	2.4	341
15	9.7	0.5	24.7	4.1	3.3	1.4	43.7	80.3	10.3	274	2.7	329
20	10.3	0.5	27.9	2.3	3.7	1.2	45.9	84.7	5.9	216	2.7	313

[a] Reaction conditions: birch wood (250 mg), RuN/ZnO/C, MeOH (15 mL), 220 °C, H_2 (3 MPa at 25 °C), and 4 h. [b] TON denotes turnover numbers, calculated based on the total number of moles of Ru in the catalyst ($mol_{phenols}$ mol_{Ru}^{-1}). [c] N.D. refers to not detected.

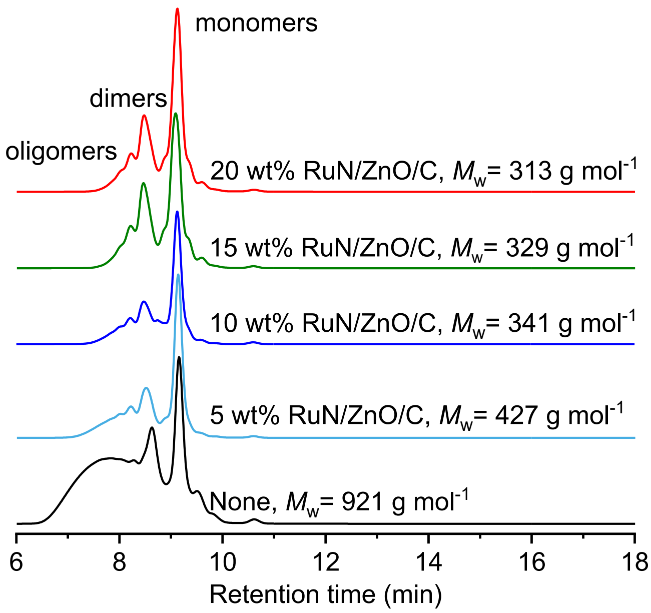

Supplementary Fig. 18 GPC spectra of lignin-derived products with different RuN/ZnO/C catalyst dosage. Reaction conditions: birch wood (250 mg), RuN/ZnO/C, MeOH (15 mL), 220 °C, H_2 (3 MPa at 25 °C), and 4 h.

Supplementary Table 10. Products distribution of RuN/ZnO/C-catalyzed large-scale RCF reaction of birch chips with reaction time.

Time (h)	Phenolic monomers yield (wt%)							S/G	M_w (g/mol)
	**Pr-G**	**Pe-G**	**Pr-S**	**Pe-S**	**POH-S**	Others	Total		
1	0.7	1.4	0.8	2.6	N.D.	0.3	5.6	1.5	547
1.5	1.1	2.2	0.9	5.1	0.1	0.6	10.0	1.6	534
2	1.5	2.5	1.3	6.4	0.3	0.9	12.9	1.7	-
2.5	1.8	2.6	1.6	7.6	0.3	1.0	14.9	1.8	504
3	2.3	2.7	2.2	8.6	0.4	1.2	17.4	1.9	486
3.5	2.7	2.6	2.6	9.4	0.5	1.4	19.2	2.0	479
4	3.1	2.5	3.2	10.0	0.6	1.5	20.9	2.1	474
4.5	3.9	2.2	4.5	10.8	0.7	1.7	23.8	2.1	465
5	4.3	2.0	5.2	11.1	0.8	1.8	25.2	2.2	455
5.5	4.9	1.8	5.9	10.8	0.8	1.8	26.0	2.2	453
6	5.0	1.7	6.6	10.7	0.9	1.8	26.7	2.2	450
6.5	5.4	1.6	7.3	11.0	0.9	1.8	28.0	2.2	443
7	5.7	1.5	8.0	10.8	1.0	1.8	28.8	2.2	437
7.5	6.1	1.5	8.7	10.4	1.1	1.8	29.6	2.2	435
8	6.3	1.1	9.6	10.5	1.1	1.8	30.4	2.2	433
8.5	6.5	1.4	10.0	10.5	1.2	1.7	31.3	2.2	431
9	6.7	1.0	10.6	10.0	1.2	1.8	31.3	2.2	428
9.5	7.1	0.8	11.5	9.7	1.1	1.7	31.9	2.2	420
10	7.3	0.7	12.0	9.3	1.2	1.7	32.2	2.2	413
11	7.7	0.6	13.2	8.9	1.2	1.6	33.2	2.2	411

Supplementary Note 8. A large-scale RCF reaction of birch was performed in a parr autoclave (300 mL) equipped with a mechanical stirrer, and a constant pressure complementary H_2 device. The reactor was loaded with birch chips (3 g), RuN/ZnO/C catalyst (300 mg), MeOH (150 mL), and 1,3,5-trimethoxybenzene (75 mg, internal standard). At room temperature, the H_2 pressure was set at 3 MPa. Then the reactor was heated to 200 °C with stirring at 300 r.p.m, and the H_2 pressure kept in 7.2 MPa. An aliquot was taken every 30 min and analysed by GC and GPC.

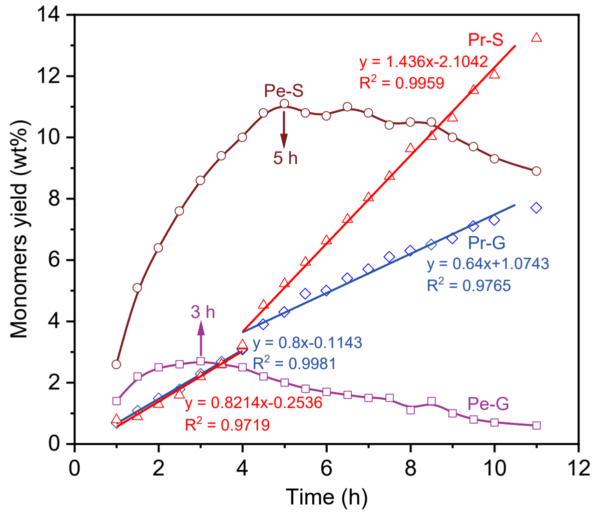

Supplementary Fig. 19 Lignin-derived products distribution from RCF of birch over RuN/ZnO/C for reaction time. Cumulative monomer yields of Pr-G, Pr-S, Pe-G and Pe-S.

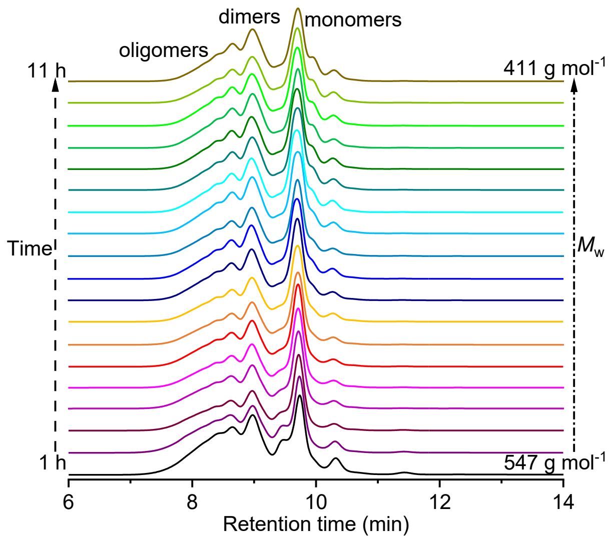

Supplementary Fig. 20 GPC spectra of lignin-derived products for reaction time in a large-scale RCF reaction. Reaction conditions: birch wood (3 g), RuN/ZnO/C (300 mg, 10 wt%), MeOH (150 mL), 1,3,5-trimethoxybenzene (75 mg, internal standard), 200 °C, and H_2 (3 MPa at 25 °C).

Supplementary Table 11. Stability and reusability of RuN/ZnO/C catalyst.[a]

Run time	Phenolic monomers yield (wt%)							Selectivity (Pr–G, Pr–S) (mol%)	Selectivity (Pe–G, Pe–S) (mol%)	TON[b]	S/G	M_w (g/mol)
	**Pr-G**	**Pe-G**	**Pr–S**	**Pe-S**	**POH–S**	Others	Total					
1st	11.1	N.D.[c]	27.9	N.D.	3.5	3.9	46.4	84.7	0	431	2.4	325
2nd	8.5	1.6	18.8	3.4	2.3	1.9	36.5	75.4	14.2	346	2.2	375
3rd	10.6	0.9	26.6	2.9	2.3	1.5	44.8	83.7	8.7	424	2.4	329
4th	9.9	0.9	22.8	2.4	3.2	1.8	41.0	80.8	8.2	388	2.3	342
5th	9.8	0.8	20.7	3.1	4.0	1.7	40.1	77.8	9.5	376	2.3	399
H.T.	10.8	N.D.	27.5	N.D.	3.1	2.7	44.1	88.2	0	414	2.4	337

[a] Reaction conditions: birch wood (250 mg), RuN/ZnO/C (25 mg, 10 wt%), MeOH (15 mL), 240 °C, H_2 (3 MPa at 25 °C), and 4 h. [b] TON denotes turnover numbers, calculated based on the total number of moles of Ru in the catalyst ($mol_{phenols}$ mol_{Ru}^{-1}). [c] N.D. refers to not detected. 1st refers to the fresh catalyst. For the 2nd run, the recovered catalyst was directed used after simple washing and drying. For 3rd, 4th and 5th runs, the recovered catalysts were calcinated at 500 °C for 2 h under N_2 flow. H.T. denotes RuN/ZnO/C after hydrothermal treatment (200 °C, 72 h).

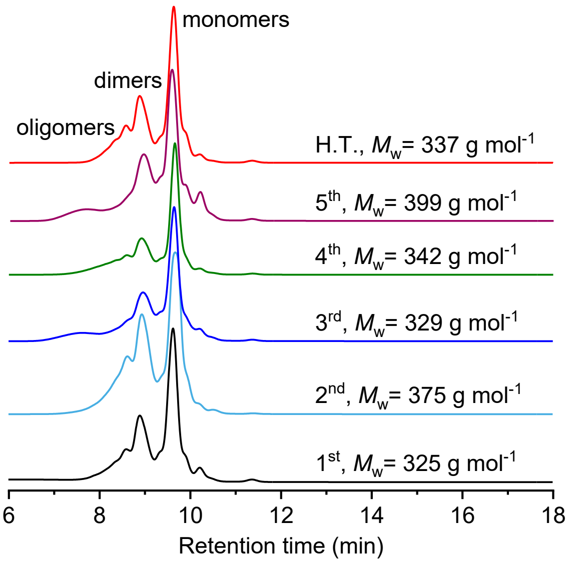

Supplementary Fig. 21 GPC spectra of lignin-derived products by using different treated RuN/ZnO/C catalyst. Reaction conditions: birch wood (250 mg), RuN/ZnO/C (25 mg, 10 wt%), MeOH (15 mL), 240 °C, H_2 (3 MPa at 25 °C), and 4 h.

Supplementary Table 12. Products distribution of RuN/ZnO/C-catalyzed RCF reaction of various biomass.[a]

Biomass	Phenolic monomers yield (wt%)							Selectivity (**Pr–G, Pr–S**) (mol%)	TON[b]	S/G	M_w (g/mol)
	**Pr-G**	**Pe-G**	**Pr–S**	**Pe-S**	**POH–S**	Others	Total				
Beech	8.7	0.5	28.8	0.2	1.8	1.5	41.5	91.6	413	3.2	371
Eucalyptus	12.4	0.6	25.0	0.2	1.7	2	41.9	90.1	435	1.9	604
Poplar	7.6	0.8	23.9	0.4	1.6	3.6	37.9	85.3	350	2.6	354
Pine	12.4	0.4	N.D. [c]	N.D.	N.D.	1.5	14.3	86.7	223	-	430
Spruce	12.1	1.5	N.D.	N.D.	N.D.	2.6	16.2	74.9	274	-	484
Miscanthus	4.2	0.7	5.9	0.3	0.8	14.8[d]	26.7	36.4	309	-	544

[a] Reaction conditions: biomass chips (250 mg), RuN/ZnO/C (25 mg, 10 wt%), MeOH (15 mL), 240 °C, H_2 (3 MPa at 25 °C), and 4 h. [b] TON denotes turnover numbers, calculated based on the total number of moles of Ru in the catalyst ($mol_{phenols}\ mol_{Ru}^{-1}$). [c] N.D. refers to not detected. [d] Containing two specific phenolic monomers (**CA-H**, 6.2 wt%; **FA-H**, 3.8 wt%) from the *p*-coumaric and ferulic acid units.

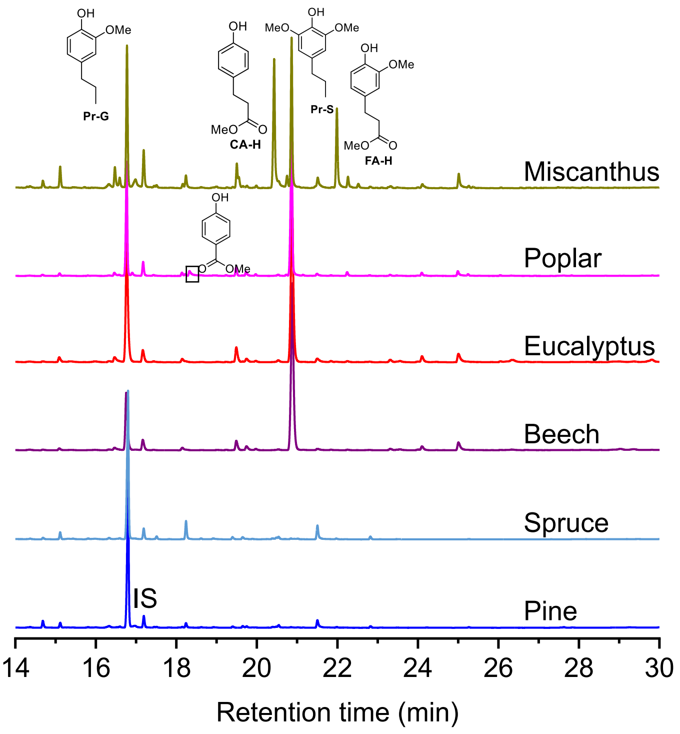

Supplementary Fig. 22 GC spectra of the lignin-derived monomers from various biomass over RuN/ZnO/C. Reaction conditions: biomass chips (250 mg), RuN/ZnO/C (25 mg, 10 wt%), MeOH (15 mL), 240 °C, H_2 (3 MPa at 25 °C), and 4 h.

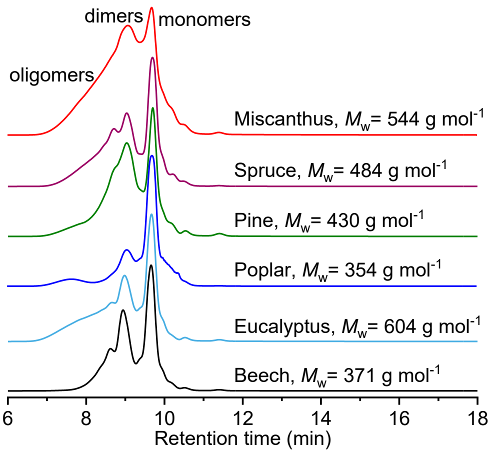

Supplementary Fig. 23 GPC spectra of lignin-derived products from various biomass over RuN/ZnO/C. Reaction conditions: biomass chips (250 mg), RuN/ZnO/C (25 mg, 10 wt%), MeOH (15 mL), 240 °C, H_2 (3 MPa at 25 °C), and 4 h.

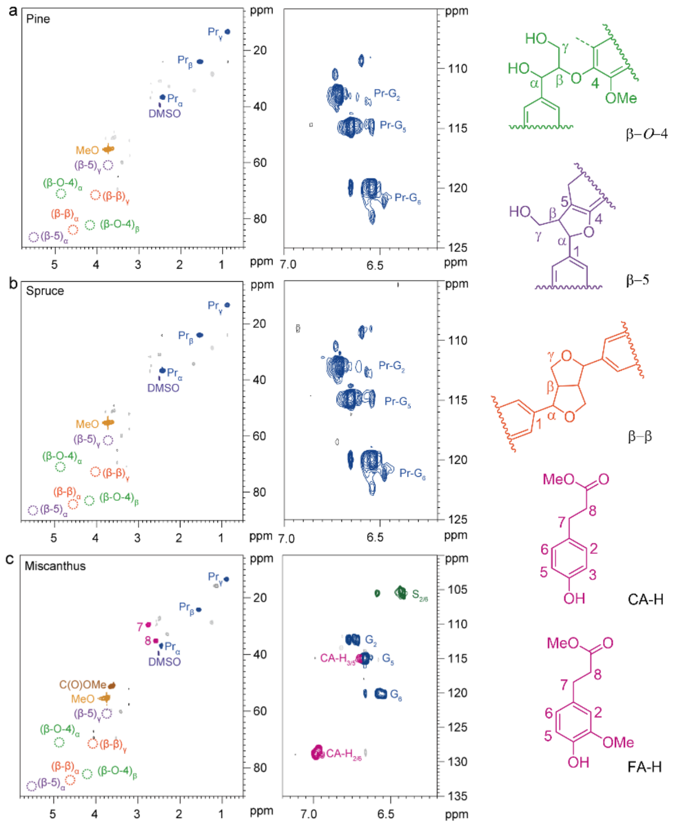

Supplementary Fig. 24 2D HSQC NMR spectra (DMSO-d_6) of lignin oil product derived from various sawdust *via* RCF over RuN/ZnO/C. (**a**) pine, (**b**) spruce, and (**c**) miscanthus.

Catalyst Recuperation and Carbohydrates Analyses

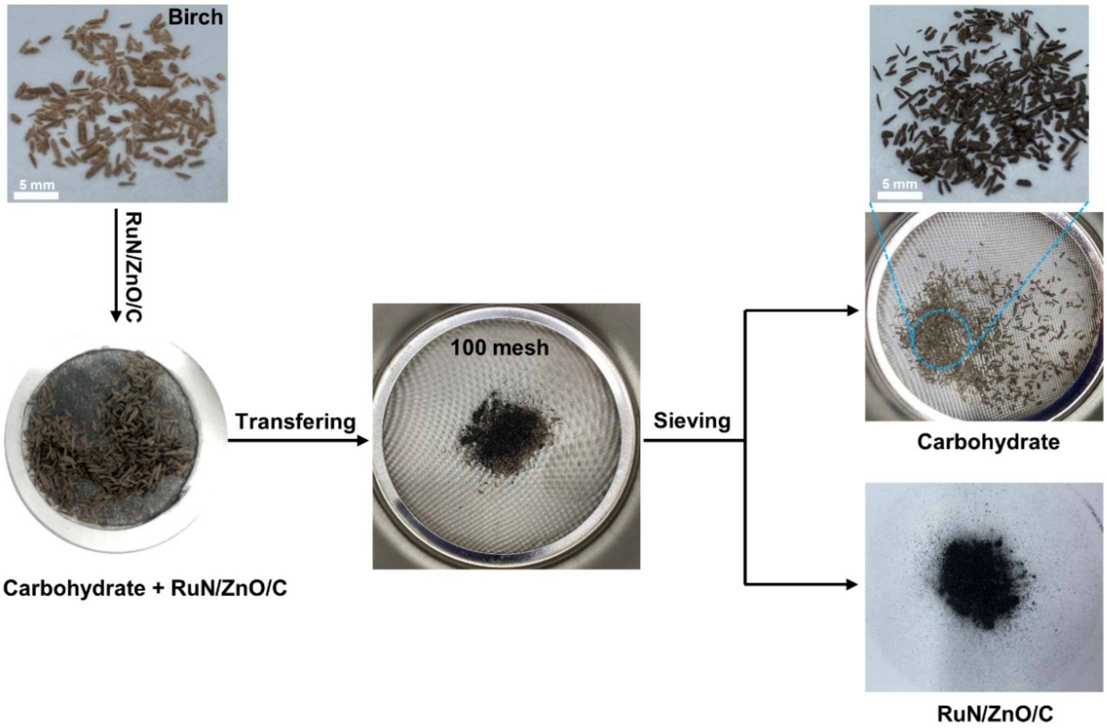

Supplementary Fig. 25 The flow-process diagram of separation the catalyst and carbohydrate. Separation the catalyst from carbohydrates. After RCF reaction, the solid phase containing carbohydrate pulp and catalyst was dried at room temperature, which was then transferred to a 100-mesh screening. The spent catalyst could be readily separated from the carbohydrate pulp by sieving, because the carbohydrate remained the original framework of biomass (2-5 mm) without collapse.

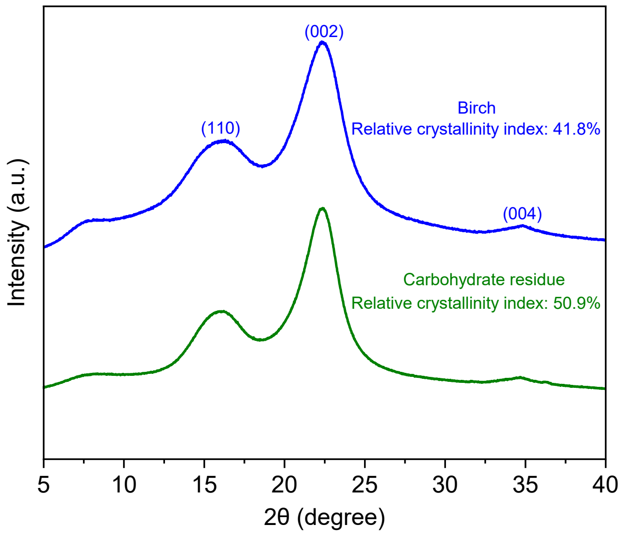

Supplementary Fig. 26 XRD profiles of birch and carbohydrate residue. Carbohydrate residue derived from RCF reaction: birch wood (250 mg), RuN/ZnO/C (25 mg, 10 wt%), MeOH (15 mL), 240 °C, H_2 (3 MPa at 25 °C), and 4 h.

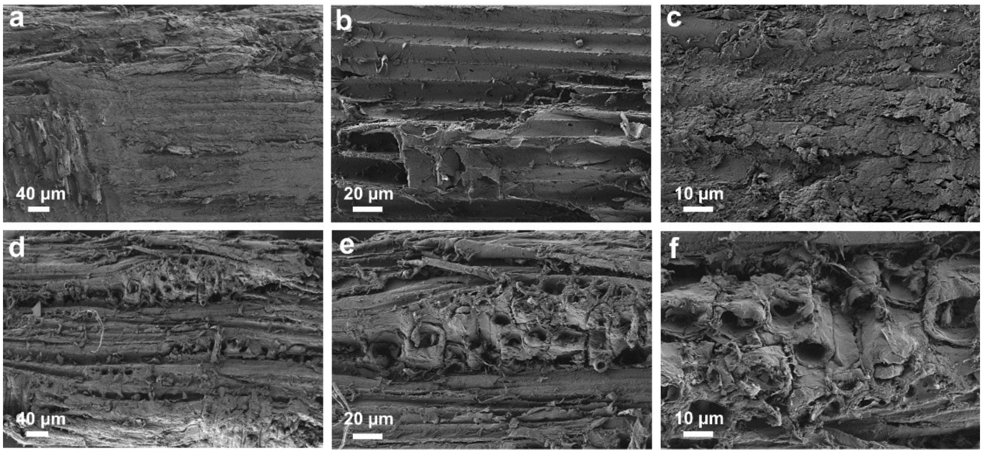

Supplementary Fig. 27 SEM images of different samples. (**a-c**) birch wood, (**d-f**) carbohydrate residue. Reaction conditions: birch wood (250 mg), RuN/ZnO/C (10 wt%), MeOH (15 mL), H_2 (3 MPa at 25 °C), 240 °C, and 4 h.

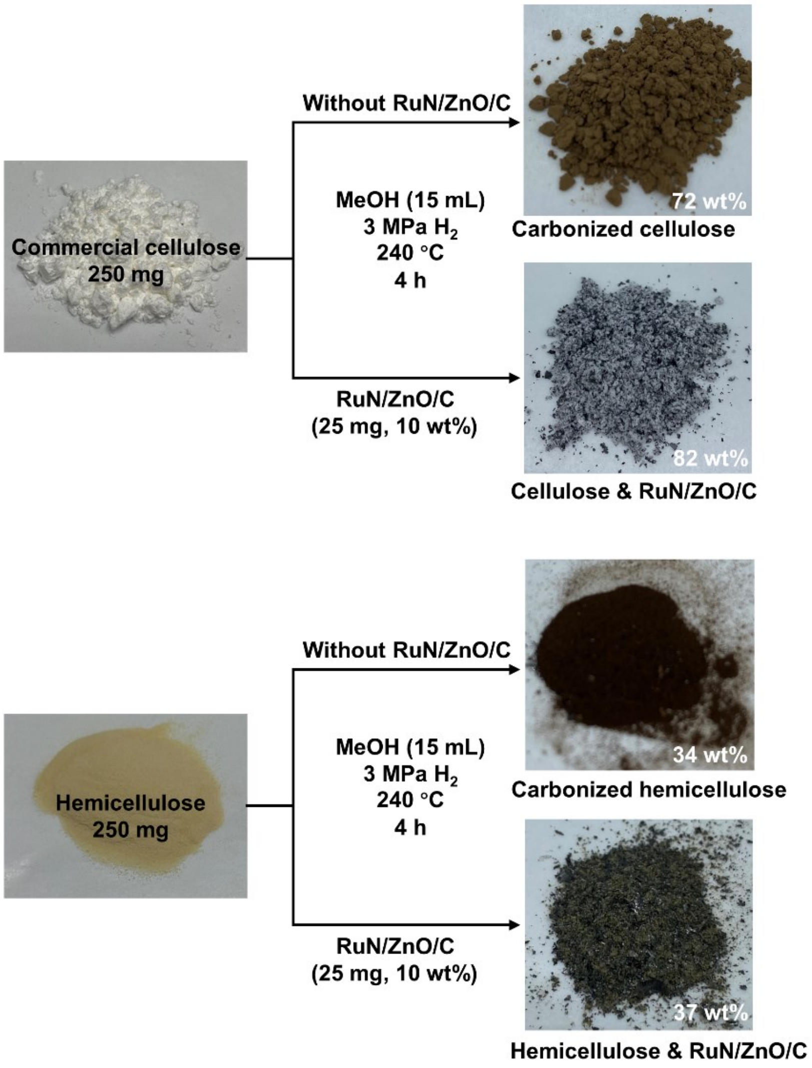

Supplementary Fig. 28 The reactivity of carbohydrate with RuN/ZnO/C. Independent experiments using commercial cellulose and hemicellulose with or without RuN/ZnO/C.

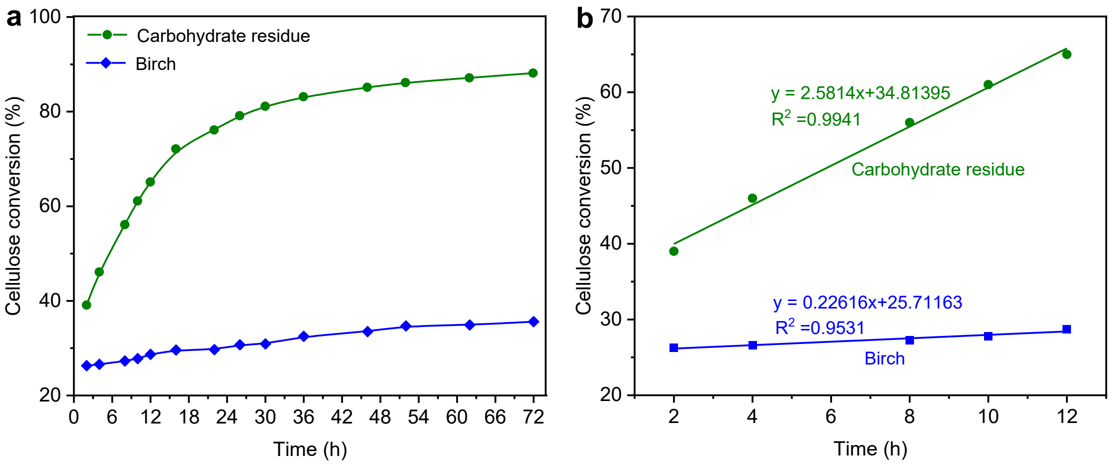

Supplementary Fig. 29 The enzymatic hydrolysis results of birch and carbohydrate residue. (**a**) Reaction time is 72 h. (**b**) Reaction time is 12 h.

Supplementary Note 9. Enzymolysis of carbohydrate residue. 500 mg of carbohydrates pulp, 20 mL of citric acid buffer (pH = 4.8, 50 mM), and 1 mL of cellulase were added in the shaking flask, which placing in an incubator shaker at 50 °C under shaking at 150 r.p.m. At every few hour intervals, 0.1 mL of reaction sample was taken out, inactivated enzyme, dilution and filtered with a 0.22 µm filter. The samples were further evaluated using HPLC system (Shimadzu LC-20AD) through a Bio-Rad Aminex HPX-87H column with 5 mM H_2SO_4 solution as mobile phase. Birch sawdust was also conducted as control enzymatic hydrolysis experiment.

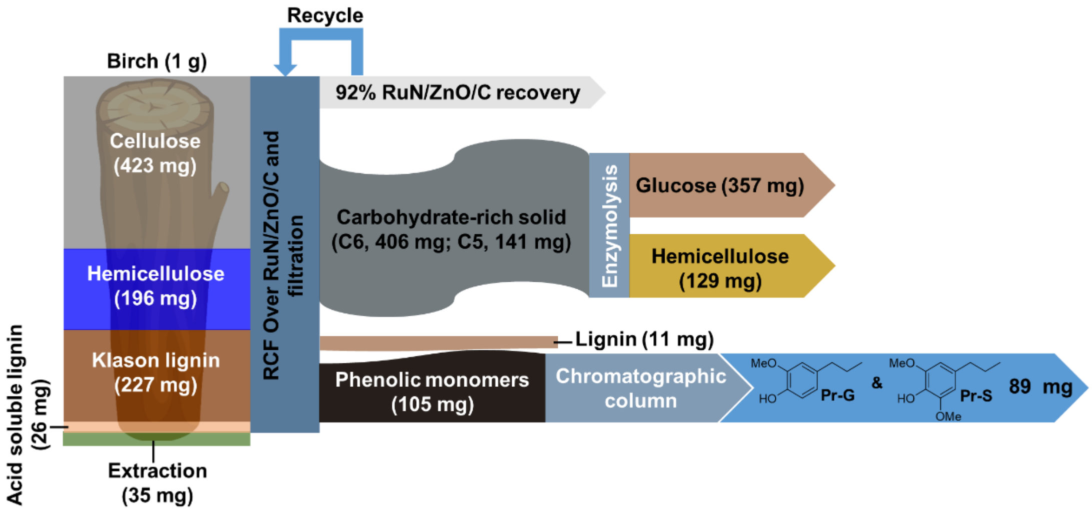

Supplementary Fig. 30 The composition of birch and mass balance of RCF reaction over RuN/ZnO/C. Reaction conditions: birch chips (1 g), RuN/ZnO/C (100 mg, 10 wt%), MeOH (60 mL), H_2 (3 MPa at 25 °C), 240 °C, and 4 h.

Reactivity of Lignin Model Compounds

General experimental procedure: In a 50 mL autoclave with a magnetic stirring bar, lignin model compound (25 mg), RuN/ZnO/C (5 mg, 20 wt%), and MeOH (15 ml) were charged and sealed. The sealed autoclave was evacuated and filled with H_2 (3 MPa) at room temperature. After stirring at 220 °C for 4 h, the autoclave was cooled to room temperature and depressurized carefully. The reaction mixture was filtered and the filtrates were evaporated to afford an oily product. The oily product was dissolved into ethyl acetate containing a standard (tetradecane) in a 5 mL volumetric flask, which was then submitted to GC and GC-MS. The identification and quantification of products were assessed by comparison with authentic samples.

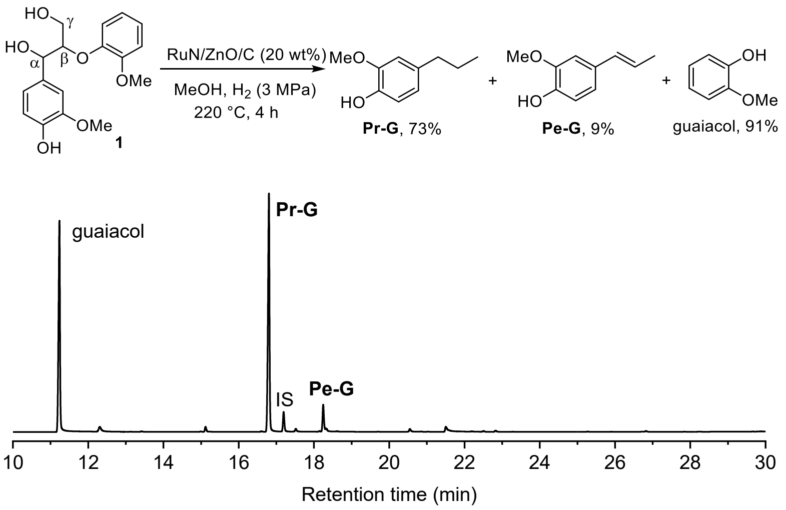

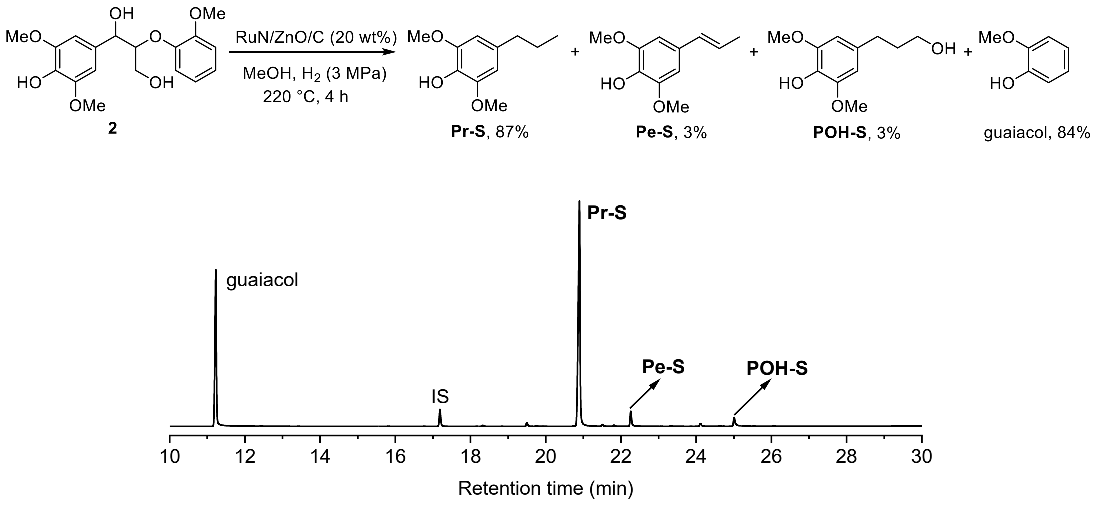

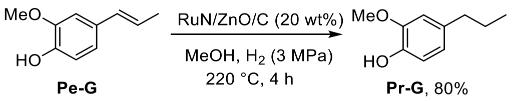
MeO
HO
Pe-G
RuN/ZnO/C (20 wt%)
MeOH, H_2 (3 MPa)
220 °C, 4 h
MeO
HO
Pr-G, 80%

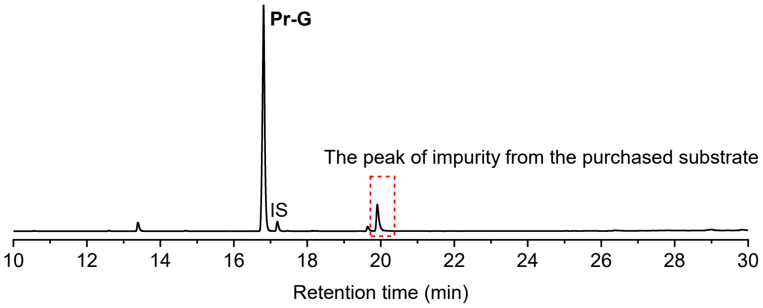
Pr-G
The peak of impurity from the purchased substrate
IS
10 12 14 16 18 20 22 24 26 28 30
Retention time (min)

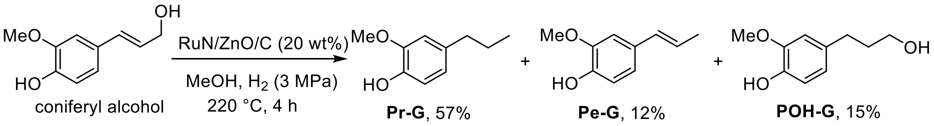
OH
MeO
HO
coniferyl alcohol
RuN/ZnO/C (20 wt%)
MeOH, H_2 (3 MPa)
220 °C, 4 h
MeO
HO
Pr-G, 57%
+
MeO
HO
Pe-G, 12%
+
MeO
OH
HO
POH-G, 15%

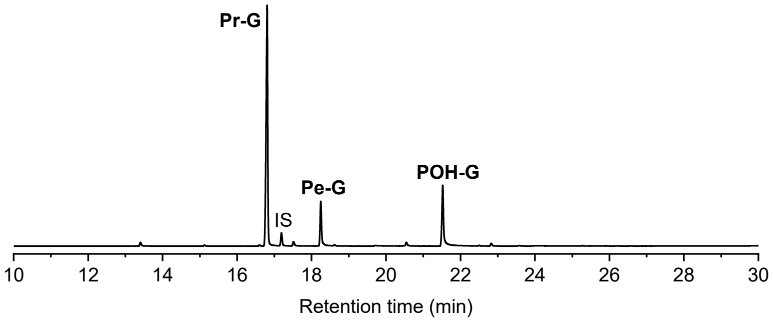
Pr-G
Pe-G
POH-G
IS
10 12 14 16 18 20 22 24 26 28 30
Retention time (min)

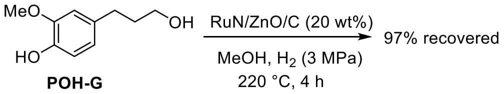
MeO
OH
HO
POH-G
RuN/ZnO/C (20 wt%)
MeOH, H_2 (3 MPa)
220 °C, 4 h
97% recovered

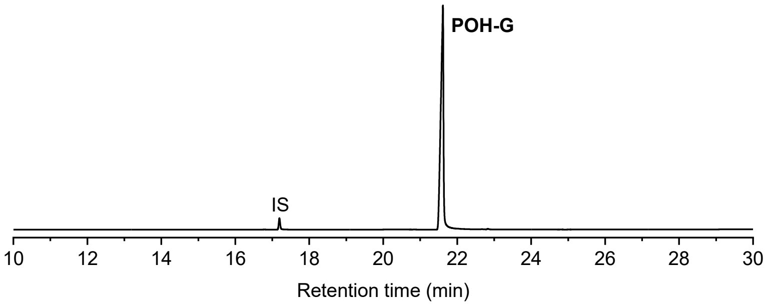
POH-G
IS
10 12 14 16 18 20 22 24 26 28 30
Retention time (min)

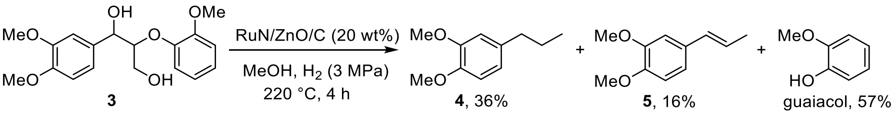
OH
OMe
MeO
MeO
OH
3
RuN/ZnO/C (20 wt%)
MeOH, H_2 (3 MPa)
220 °C, 4 h
4, 36%
5, 16%
HO
guaiacol, 57%

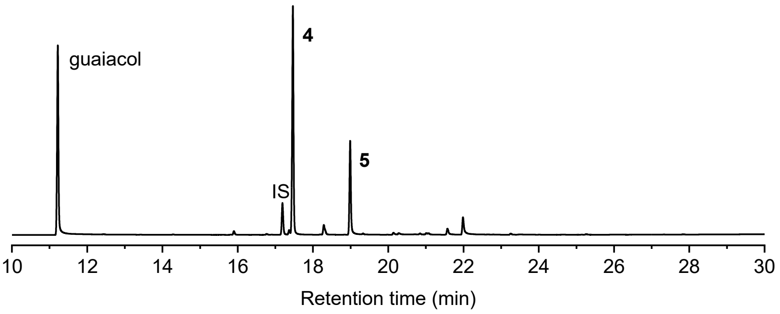
guaiacol
4
IS
5
10
12
14
16
18
20
22
24
26
28
30
Retention time (min)

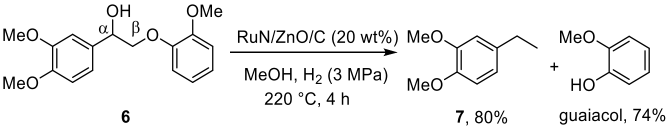
OH
OMe
α
β
MeO
MeO
6
RuN/ZnO/C (20 wt%)
MeOH, H_2 (3 MPa)
220 °C, 4 h
7, 80%
HO
guaiacol, 74%

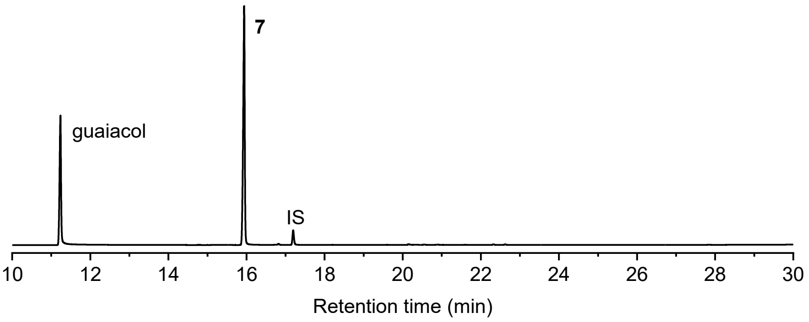
7
guaiacol
IS
10
12
14
16
18
20
22
24
26
28
30
Retention time (min)

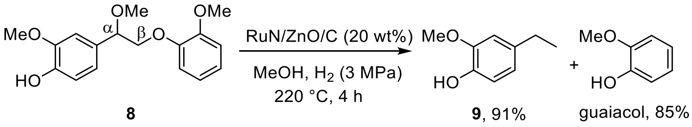
OMe
OMe
α
β
MeO
HO
8
RuN/ZnO/C (20 wt%)
MeOH, H_2 (3 MPa)
220 °C, 4 h
9, 91%
guaiacol, 85%

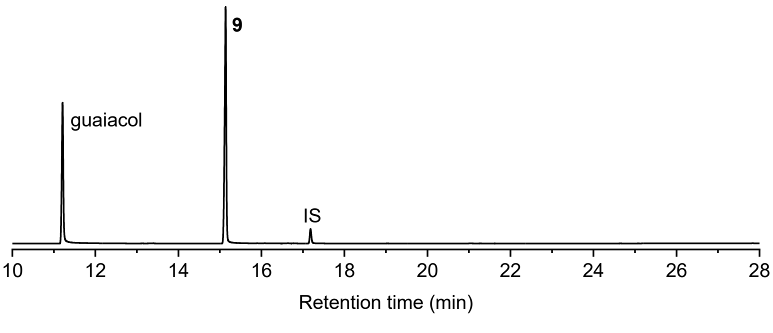
9
guaiacol
IS
10
12
14
16
18
20
22
24
26
28
Retention time (min)

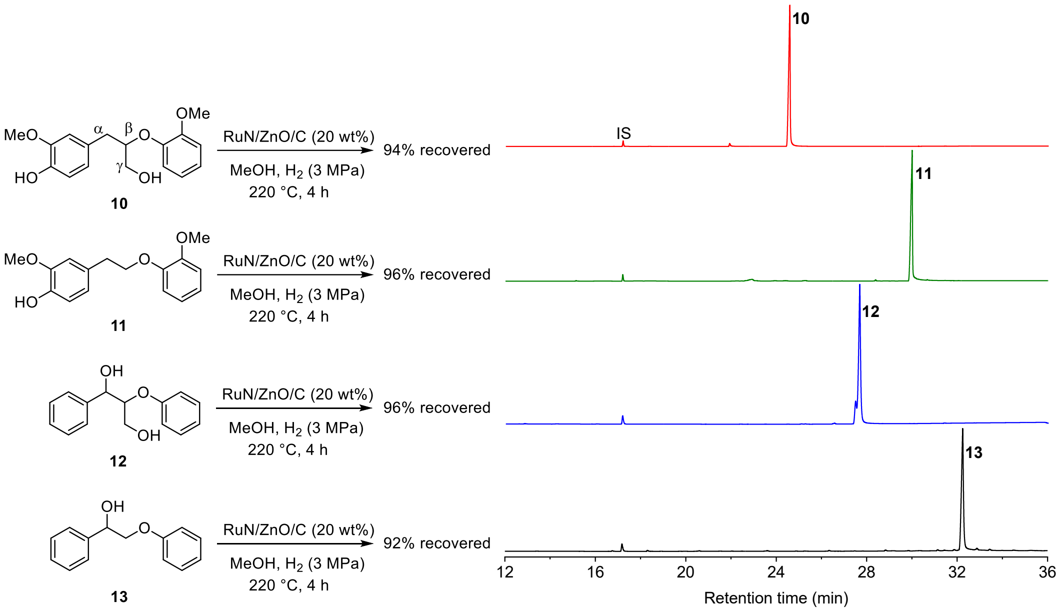

Supplementary Fig. 31 RuN/ZnO/C-catalyzed hydrogenolysis of various lignin model compounds. Model compounds **1** and **2** refer phenolic β-*O*-4 dimers. Model compound **3** refers nonphenolic β-*O*-4 dimers. Model compounds **6** and **8** refer β-*O*-4 dimers lacking of γ-CH_2OH unit. Model compounds **10**, **11**, **12** and **13** refer nonreactive β-*O*-4 dimers over RuN/ZnO/C catalyst.

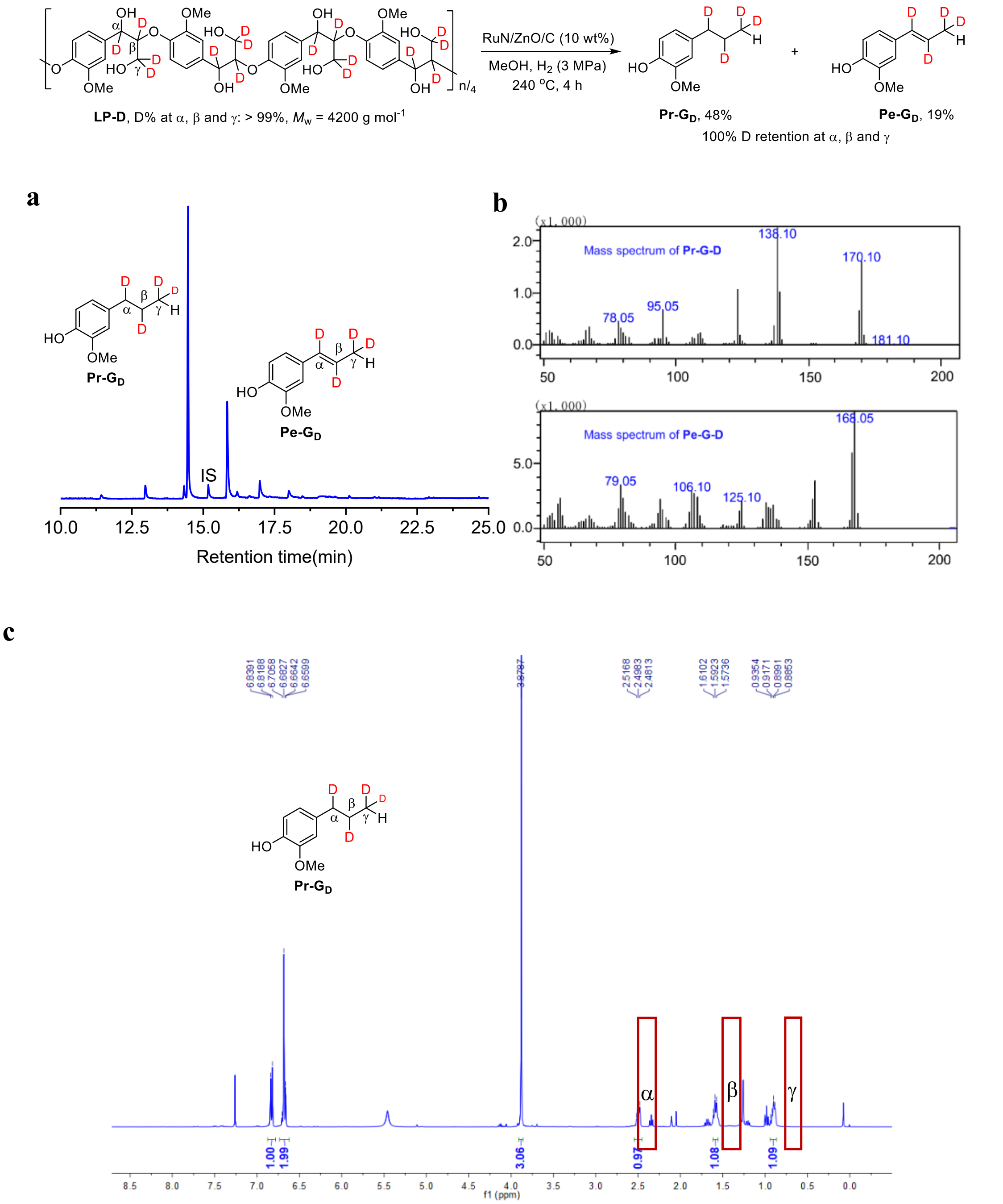

Supplementary Fig. 32 Hydrogenolysis of D-incorporated β-*O*-4 polymer LP-D with RuN/ZnO/C. (**a**) GC spectrum. (**b**) Mass spectrum derived from GC-MS. (**c**) ^{1}H NMR spectrum of isolated Pr-GD.

Supplementary Note 10. Compound Pr-GD was separated and purified through chromatographic column (PE/EA), and further characterized by NMR. ^{1}H NMR and mass analyses indicated that the deuteriums at α, β and γ positions remained intact.

Reported RCF Literatures

Supplementary Table 13. Comparison of reductive catalytic fractionation results over different catalyst in literatures.

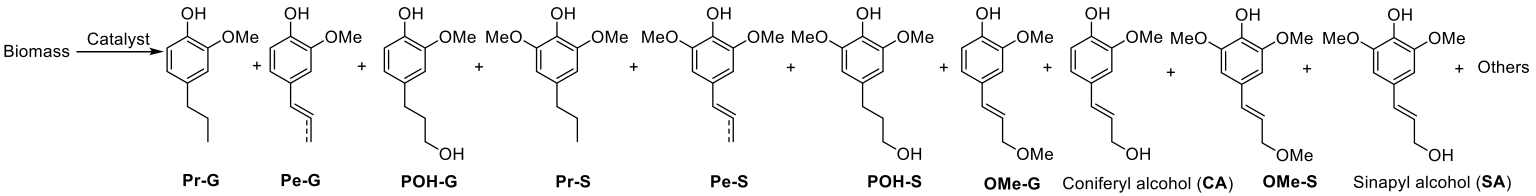

Catalyst	Reaction conditions	Primary monomers	Total monomers yield	Delignification	Sugar retention	TON[a]	Reference
Pd/CN_x (Pd, 0.25 wt%)	Birch (3 g) Pd/CN_x (0.45 g), MeOH (60 mL), 250 °C, 3 MPa H_2, 3 h	Pr-G; Pr-S Pe-G; POH-G POH-S	52.7 C%	87.1 wt%	84.2 wt%	169	8
Co-phen/C (Co, 0.45 wt%)	Birch (0.2 g), Co-phen/C (0.03 g), EtOH/H_2O (6 mL, 1:1 v/v), 200 °C, 4 h HCOOH + HCOONa (5+5)	Pr-G; Pr-S Pe-G; Pe-S	34 wt%	-	-	100	9
Pt_1Ni/C (Pt, 0.3 wt%; Ni, 4.4 wt%)	Birch (0.1 g), Pt_1Ni/C (0.02 g), MeOH (5 mL), 300 °C, 5 MPa H_2, 18 h	Pr-G; Pr-S	43 wt%	-	-	166	10
Ru/C (Ru, 5 wt%)	Birch (2 g), Ru/C (0.3 g), MeOH (40 mL), 250 °C, 3 MPa H_2, 6 h	Pr-G; Pr-S	52 C%	92 wt%	C6 (95 C%) C5 (47 C%)	7	4

Continued

MoO_x/SBA-15<br>(Mo, 5.7 wt%)	Eucalyptus (0.5 g), MoO_x/CNT (0.05 g),<br>MeOH (15 mL), 260 °C, 3 MPa H_2, 4 h	OMe-G, OMe-S<br>CA, SA	43.4 wt%	95 wt%	C6 (98 wt%)<br>C5 (89 C%)	8	11
Ni/C<br>(Ni, 10 wt%)	Birch (2 g), Ni/C (0.1 g),<br>MeOH (40 mL), 200 °C, 0.1 MPa A_r, 6 h	Pr-G; Pr-S	54 wt%	-	-	6	12
Ni@ZIF-8<br>(Ni, 14 wt%)	Eucalyptus (0.5 g), 260 °C, 3 MPa H_2, 8 h<br>Ni@ZIF-8 (0.05 g), MeOH (10 mL),	Pr-G; Pr-S<br>POH-G; POH-S	44.3 wt%	92 wt%	C6 (90 wt%)<br>C5 (67 wt%)	2	13
Ni_1-Fe_1/AC<br>(Ni, 5 wt%)	Birch (0.5 g), Ni_1-Fe_1/AC (0.05 g),<br>MeOH (10 mL), 200 °C, 2 MPa H_2, 6 h	Pr-G; Pr-S	39.5 wt%	-	-	5	14
Pd/C<br>(Pd, 5 wt%)	Birch (2 g), Pd/C (0.2 g),<br>Al(III) triflate (0.0322 mmol)<br>MeOH (40 mL), 180 °C, 3 MPa H_2, 2 h	OMe-G, OMe-S<br>Pr-G; Pr-S<br>POH-G; POH-S	44 wt%	81 wt%	C6 (97 wt%)<br>C5 (44 wt%)	12	15
Cu20-PMO<br>(Cu, 15.4 wt%)	Poplar (1 g), Cu20-PMO (0.2 g),<br>MeOH (10 mL), 180 °C, 4 MPa H_2, 18 h	Pr-G; Pr-S<br>POH-G; POH-S	36 wt%	-	-	0.7	16
RuN/ZnO/C<br>(Ru, 0.12 wt%; Zn, 6.2 wt%)	Birch (0.25 g), RuN/ZnO/C (0.025 g),<br>MeOH (15 mL), 240 °C, 3 MPa H_2, 4 h	Pr-G; Pr-S	46.4 wt%	95 wt%	C6 (96 wt%)<br>C5 (72 C%)	431	This<br>work

[a]TON denotes turnover numbers, calculated based on the total number of moles of active metal in the catalyst ($mol_{phenols}\ mol_{Active\ metal}^{-1}$)

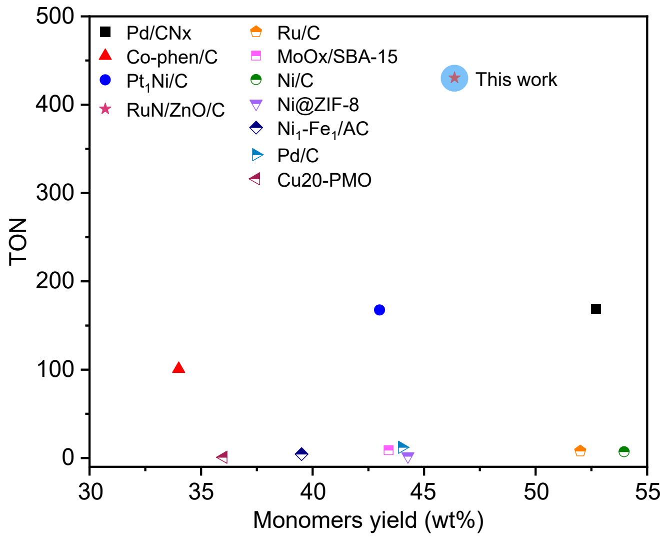

Supplementary Fig. 33 Reported TON of reductive catalytic fractionation results over different catalyst. In this work, the TON is calculated as 431 $mol_{phenols}$ mol_{Ru}^{-1} based on the total number of moles of Ru in RuN/ZnO/C, which is significantly larger than those from other supported metal catalysts.

Standard Curves

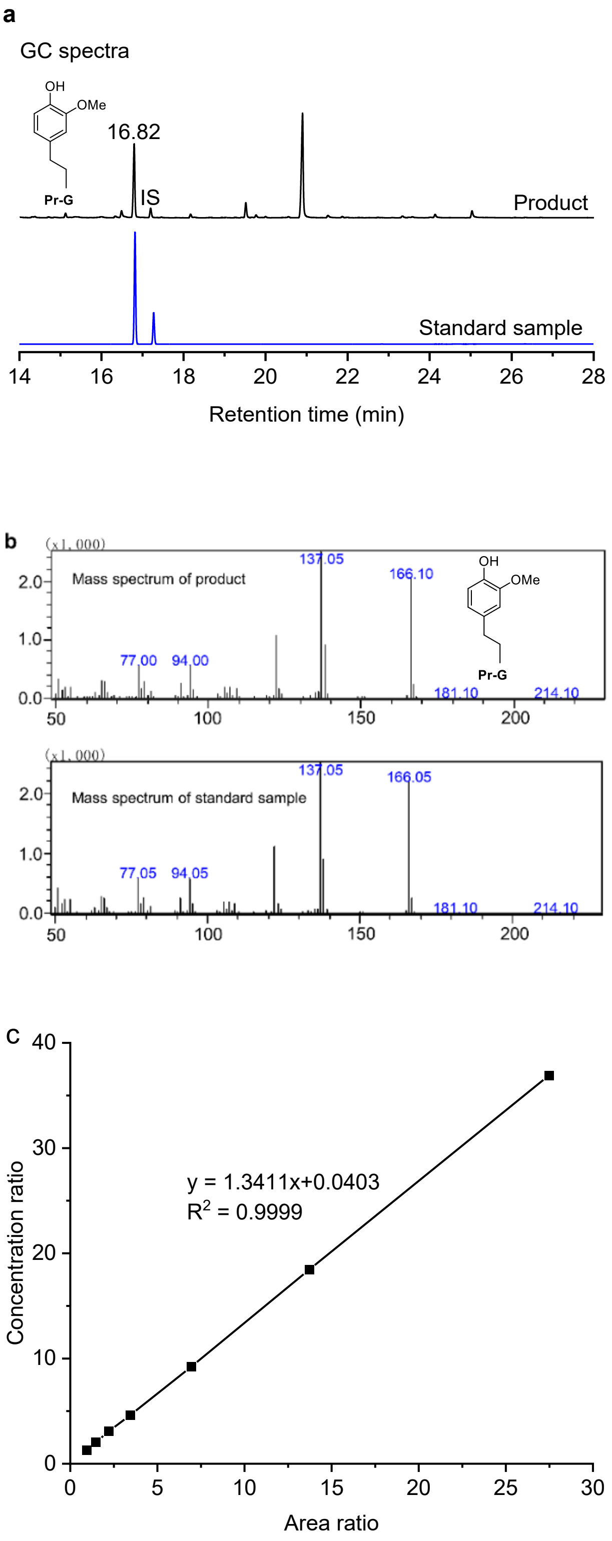

Supplementary Fig. 34 Qualitative and quantitative analyses of Pr-G. (**a**) GC, (**b**) mass spectra of Pr-G derived from birch chips depolymerization and standard sample Pr-G. (**c**) Standard curve of Pr-G.

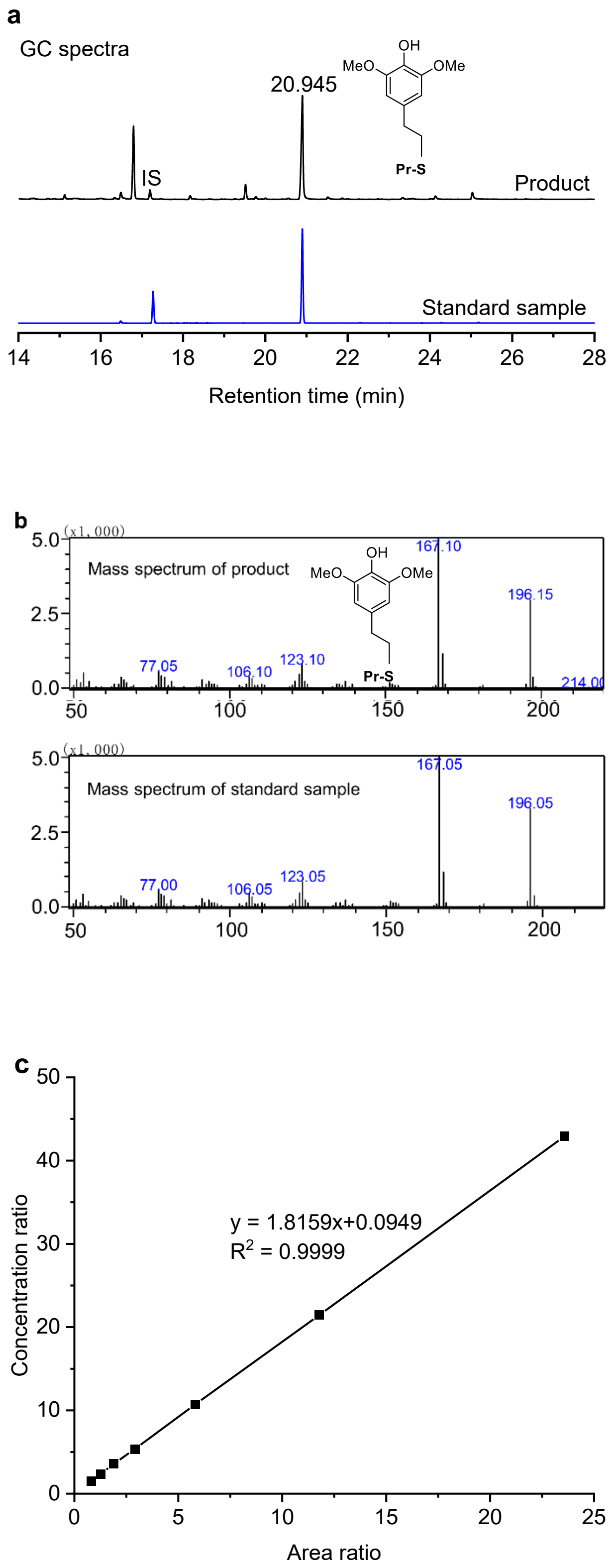

Supplementary Fig. 35 Qualitative and quantitative analyses of Pr-S. (**a**) GC, (**b**) mass spectra of Pr-S derived from birch chips depolymerization and standard sample Pr-S. (**c**) Standard curve of Pr-S.

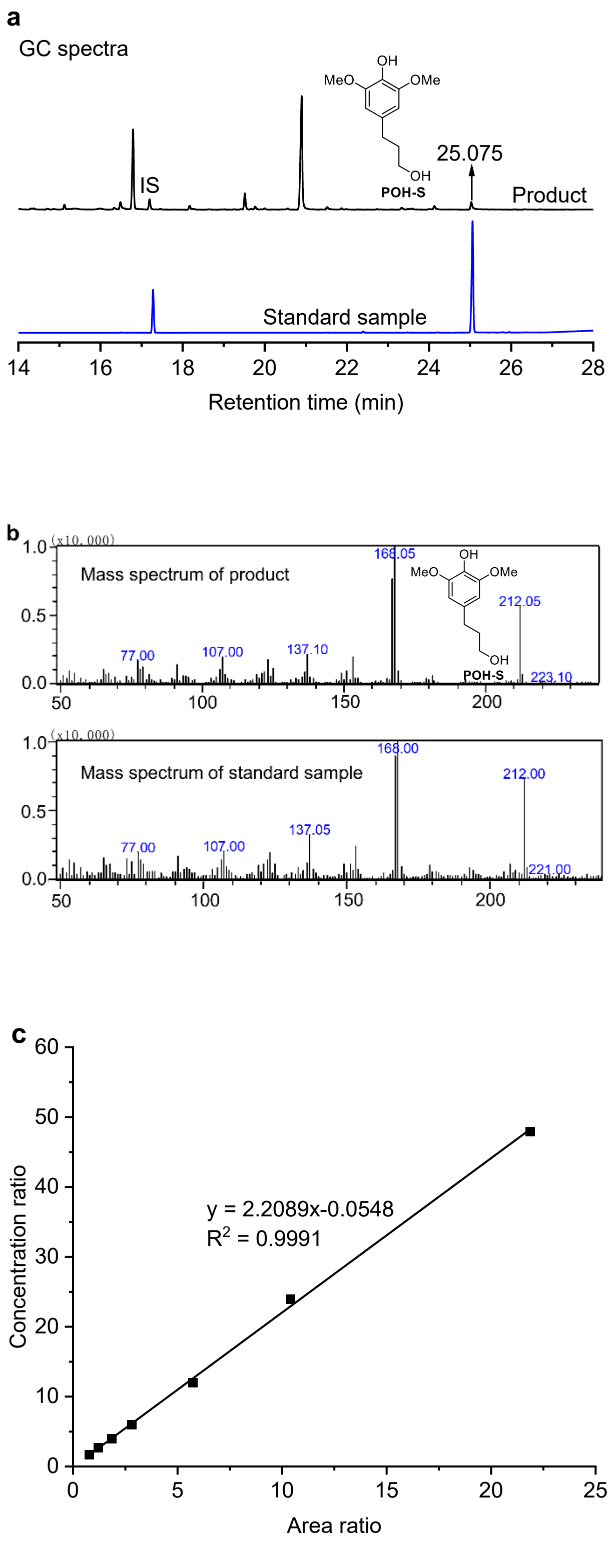

Supplementary Fig. 36 Qualitative and quantitative analyses of POH-S. (**a**) GC, (**b**) mass spectra of POH-S derived from birch chips depolymerization and standard sample POH-S. (**c**) Standard curve of POH-S.

Supplementary References

1. Chen, W. et al. Rational design of single molybdenum atoms anchored on N-doped carbon for effective hydrogen evolution reaction. *Angew. Chem. Int. Ed.* **56**, 16086-16090 (2017).
2. Ravel, B. & Newville, M. ATHENA and ARTEMIS: Interactive graphical data analysisusing IFEFFIT. *Phys. Scr.* **115**, 1007-1010 (2005).
3. Sluiter, A. et al. Determination of structural carbohydrates and lignin in biomass, National renewable energy laboratory (NREL), Golden, Colorado, 2008.
4. Van den Bosch, S. et al. Reductive lignocellulose fractionation into soluble lignin-derived phenolic monomers and dimers and processable carbohydrate pulps. *Energy Environ. Sci*. **8**, 1748-1763 (2015).
5. Anderson, E.M. et al. Flowthrough reductive catalytic fractionation of biomass. *Joule*. **1**, 613-622 (2017).
6. Anderson, E.M. et al. Differences in S/G ratio in natural poplar variants do not predict catalytic depolymerization monomer yields. *Nat. Commun*. **10**, 2033 (2019).
7. Safamirzaei, M. Modarress, H. & Mohsen-Nia, M. Modeling the hydrogen solubility in methanol, ethanol, 1-propanol and 1-butanol. *Fluid Phase Equilibr*. **289**, 32-39 (2010).
8. Park, J. et al. Highly efficient reductive catalytic fractionation of lignocellulosic biomass over extremely low-loaded Pd catalysts. *ACS Catal.* **10**, 12487-12506 (2020).
9. Rautiainen, S. et al. Lignin valorization by cobalt-catalyzed fractionation of lignocellulose to yield monophenolic compounds. *ChemSusChem* **12**, 404-408 (2019).
10. Chen, L. et al. Anchoring single platinum atoms onto nickel nanoparticles affords highly selective catalysts for lignin conversion. *Cell Reports Physical Science.* **2**, 100567 (2021).
11. Sun, J. et al. Fragmentation of woody lignocellulose into primary monolignols and their derivatives. *ACS Sustain. Chem. Eng.* **7**, 4666-4674 (2019).
12. Song, Q. et al. Lignin depolymerization (LDP) in alcohol over nickel-based catalysts via a fragmentation-hydrogenolysis process. *Energy Environ. Sci*. **6**, 994-1007 (2013).
13. Liu, X., Li, H., Xiao, L.-P., Sun, R.-C. & Song, G. Chemodivergent hydrogenolysis of eucalyptus lignin with Ni@ZIF-8 catalyst. *Green Chem.* **21**, 1498-1504 (2019).

14. Zhai, Y. et al. Depolymerization of lignin via a non-precious Ni-Fe alloy catalyst supported on activated carbon. *Green Chem.* **19**, 1895-1903 (2017).
15. Huang, X. et al. Reductive fractionation of woody biomass into lignin monomers and cellulose by tandem metal triflate and Pd/C catalysis. *Green Chem.* **19**, 175-187 (2017).
16. Sun, Z. et al. Complete lignocellulose conversion with integrated catalyst recycling yielding valuable aromatics and fuels. *Nat. Catal.* **1**, 82-92 (2018).
